# Colony-stimulating factor 3 as a key mediator in the progression of idiopathic pulmonary fibrosis: a novel therapeutic target

**DOI:** 10.1038/s41392-025-02421-6

**Published:** 2025-10-02

**Authors:** Seungmo Kim, Yongjoong Kim, Tae-Sung Kim, Jae-Hyeok Kang, In-Yeong Yun, Eung-Suk Lee, Eun Ji Lee, Rae-kwon Kim, Joo Mi Yi, Hye Sook Choi, Jin Woo Song, Young Woo Jin, Min-Jung Kim, Su-Jae Lee

**Affiliations:** 1 Fibrosis and Cancer Targeting Biotechnology (FNCT BIOTECH), Seoul, South Korea; 2 Department of Life Science, Research Institute for Natural Sciences, Hanyang University, Seoul, South Korea; 3 Department of Biotechnology, Yong In University, Yongin-si, Gyeonggi-do South Korea; 4 Department of Microbiology and Immunology, College of Medicine, Inje University, Busan, South Korea; 5 Cardiovascular and Metabolic Diseases Medical Research Center, College of Medicine, Inje University, Busan, South Korea; 6 Division of Pulmonary, Allergy and Critical Care Medicine, Department of Internal Medicine, Kyung Hee University College of Medicine, Seoul, Korea; 7 Department of Pulmonary and Critical Care Medicine, Asan Medical Center, University of Ulsan College of Medicine, Seoul, Republic of Korea

**Keywords:** Target identification, Therapeutics, Drug development, Respiratory tract diseases

## Abstract

Idiopathic pulmonary fibrosis (IPF) is a fatal lung disease characterized by excessive ECM deposition and myofibroblast accumulation driven by cytokine dysregulation. This study identified granulocyte colony-stimulating factor 3 (CSF3) as a key mediator of IPF progression. Elevated CSF3 expression was observed in the lung tissues of IPF patients. Recombinant CSF3 promoted myofibrogenesis in lung fibroblasts, whereas CSF3-deficient mice were protected from bleomycin-induced pulmonary fibrosis. Treatment with novel CSF3-neutralizing antibodies significantly restored fibrosis in IPF mice by suppressing myofibroblast differentiation and reducing ECM deposition. Here, we demonstrated a reciprocal regulatory relationship between CSF3 and TGF-β that amplifies pro-fibrotic signaling. Our mechanistic studies revealed that CSF3 acts as an upstream regulator of TGF-β, forming a positive feedback loop that significantly accelerates the fibrotic process. Knockout or neutralization of CSF3 suppressed fibrosis by reducing TGF-β levels, whereas treatment with recombinant CSF3 promoted fibrosis with increased TGF-β expression. Notably, while CSF3 inhibition reduced TGF-β expression levels, it did not decrease them below normal levels. This finding suggests that inhibiting CSF3 could simultaneously reduce fibrosis by suppressing excessive TGF-β expression while also minimizing side effects by maintaining TGF-β homeostasis. Taken together, these results provide strong evidence that CSF3 is a critical driver of IPF pathogenesis and that targeting CSF3 may provide a therapeutic strategy by modulating TGF-β signaling and restoring the ECM and cellular homeostasis.

## Introduction

Idiopathic pulmonary fibrosis (IPF) is a chronic, progressive, and irreversible interstitial lung disease that results in the gradual destruction of lung tissue.^1^ This damage is primarily driven by the accumulation of myofibroblasts and the excessive deposition of extracellular matrix (ECM) components. Consequently, IPF causes a progressive decline in lung function, which significantly contributes to increased morbidity and mortality. Although Pirfenidone and Nintedanib have been approved by the U.S. Food and Drug Administration as antifibrotic therapies, current treatment options remain limited and largely palliative.^2–4^ Pirfenidone is thought to exert antifibrotic effects by inhibiting Transforming Growth Factor-beta (TGF-β) signaling and reducing collagen synthesis, though its precise mechanism is not fully understood. Nintedanib, a multi-targeted tyrosine kinase inhibitor (TKI), blocks receptors such as VEGFR, FGFR, and PDGFR, which are involved in fibroblast proliferation and angiogenesis. While both drugs may slow disease progression, they do not reverse or halt the underlying pathological changes, underscoring the critical need for more effective therapeutic strategies.^5,6^ The poor prognosis associated with IPF demonstrates the urgency of identifying key molecular mechanisms that drive disease progression and discovering novel targets for therapeutic intervention.

TGF-β plays a critical role in the initiation and progression of pulmonary fibrosis, maintaining persistent activation in fibrotic tissues. This activation leads to the differentiation of fibroblasts into myofibroblasts, promotes ECM deposition, and contributes to tissue remodeling and scar formation.^7–9^ These processes result in impaired lung function and disease progression. Given its central role, therapeutic strategies targeting TGF-β activation and signaling pathways have been actively developed.^10^ However, owing to the widespread involvement of TGF-β in essential physiological processes, directly targeting TGF-β in IPF presents significant challenges.^11,12^ Many clinical trials have been halted because of severe side effects and insufficient inhibition of TGF-β signaling.^13–15^ Therefore, the most ideal therapeutic approach would selectively inhibit excessive TGF-β induced by pulmonary fibrosis, while preserving the basal TGF-β levels crucial for homeostasis.

Colony-stimulating factor 3 (CSF3), also known as granulocyte-colony stimulating factor (G-CSF), is a hematopoietic cytokine traditionally recognized for its role in promoting the production of granulocytes and stem cells from the bone marrow. In addition to its established role in hematopoiesis, CSF3 also regulates various immune responses, particularly the proliferation, survival, and activation of neutrophils.^16^ In addition to its production by monocytes and macrophages, CSF3 can be induced in other cell types, including fibroblasts, epithelial cells, and endothelial cells, particularly in response to inflammatory stimuli.^17^ While CSF3’s role in the immune system is well-established, emerging evidence suggests that it may also be implicated in the pathogenesis of several fibrotic diseases, including IPF.^18^ Elevated levels of CSF3 have been associated with chronic inflammatory conditions, autoimmune diseases, and certain cancers, indicating that its influence extends beyond immune regulation to modulate processes critical to tissue remodeling and fibrosis.^18,19^

In IPF, fibrosis progresses with minimal active inflammation.^20^ Neutrophils, which are typically involved in acute inflammation, persist in fibrotic tissue even in the absence of ongoing inflammation, contributing to tissue remodeling and fibrosis.^21,22^ CSF3 is known to play a role in neutrophil recruitment and survival; its elevated expression in IPF tissues may promote the persistence of neutrophils in fibrotic areas, exacerbating disease progression.^16–18^ However, the precise mechanisms by which CSF3 contributes to IPF, particularly in the absence of a classical inflammatory response, are not fully understood.

Recent studies have suggested that CSF3 may play a broader role in the development of pulmonary fibrosis, independent of its effects on neutrophils.^18^ A number of clinical studies have indicated a correlation between the administration of CSF3 and the development of pulmonary toxicity in patients with neutropenia.^23^ Furthermore, some studies indicate that CSF3, found in bronchoalveolar lavage fluid (BALF), may play a pivotal role in the progression of IPF and could serve as a potential biomarker for predicting disease prognosis.^18,24–26^ Nevertheless, at present, there is no direct evidence to suggest that CSF3 is involved in the onset or progression of pulmonary fibrosis. Further research is required to elucidate the potential mechanistic role of CSF3 in the development of IPF and to evaluate its utility as a prognostic tool.

The primary aim of this study is to investigate the functional role of CSF3 in the pathogenesis of IPF, extending beyond its established effects on neutrophils. Our data provide compelling evidence that CSF3 is a crucial mediator of pulmonary fibrosis progression. We demonstrate that CSF3 promotes myofibroblast activation and ECM deposition in lung fibroblasts, while genetic deletion of CSF3 confers protection against bleomycin-induced fibrosis. Mechanistically, we show that CSF3 acts as an upstream regulator of key fibrotic pathways, particularly through the activation of TGF-β, a central mediator of fibrosis. Moreover, our findings indicate that neutralizing CSF3 can reduce fibrosis by inhibiting TGF-β signaling, deactivating myofibroblasts, and restoring the ECM and cellular homeostasis. These findings position CSF3 as a promising therapeutic target for IPF, with the potential to improve patient outcomes by disrupting the pathological processes that underlie disease progression.

## Results

### CSF3 is elevated and correlated with fibrosis pathogenesis in pulmonary fibrosis

**Fig. 1:**
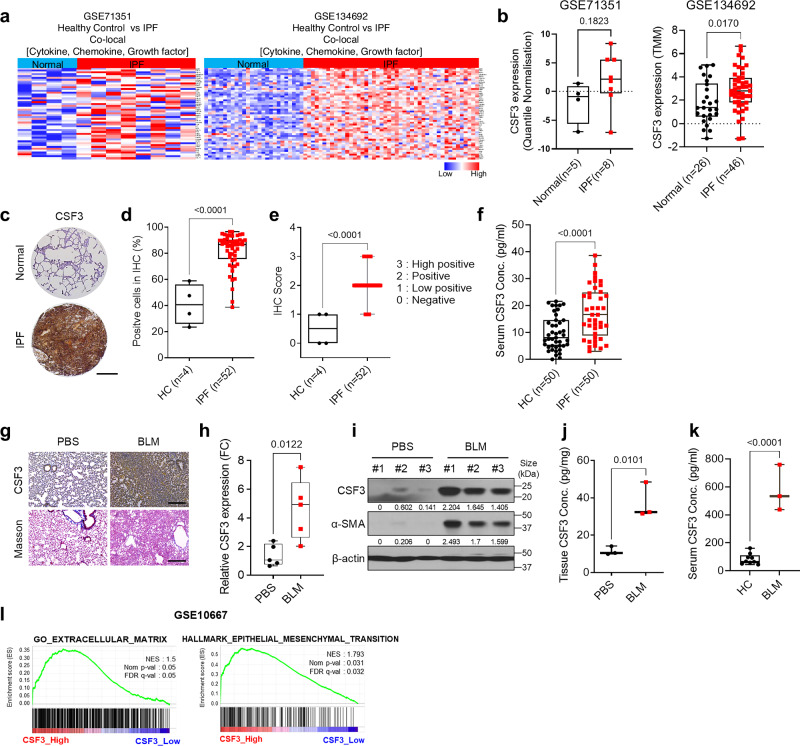
CSF3 is elevated and correlated with fibrosis pathogenesis in pulmonary fibrosis. **a** Heatmap displays gene expression in IPF patient tissues (GSE134692; *n* = 72) and patient-derived fibroblasts (GSE71351; *n* = 13) from each dataset. Cytokines, chemokines, and growth factors were selected based on a 1.5-fold increase in expression observed in IPF patients compared to healthy controls. **b** Expression levels of CSF3 in the Normal and IPF patient group (IPF) from GSE134692 and GSE71351. Each sample count is indicated in the graph. **c** Immunohistochemical staining of CSF3 in a human pulmonary fibrosis tissue array (LC561a). (Healthy control (HC) = 4, IPF = 52). Scale bar: 500 nm. **d** and **e** CSF3 immunohistochemical (IHC) scoring of a human pulmonary fibrosis tissue array, performed using two distinct IHC profiling methods. Scale bar: 200 μm. **f** Enzyme-linked immunosorbent assay (ELISA) measurement of CSF3 in human blood serum. **g** Immunostaining of CSF3 and Masson’s trichrome staining in mouse lung tissue from an intraperitoneal IPF mouse model. Scale bar = 250 μm. **h** Quantitative reverse transcription polymerase chain reaction (qRT-PCR) analysis of CSF3 expression in mouse lung tissue (*n* = 5 mice per group). **i** Western blot analysis of CSF3 and α-SMA in each group of mouse lung tissue. **j** and **k** ELISA measurements of CSF3 in mouse lung tissue (**j**) and mouse blood serum (**k**). **l** Gene set enrichment analysis (GSEA) revealed a significant correlation between extracellular matrix and epithelial-to-mesenchymal transition-related genes and IPF patients with high CSF3 expression (GSE10667). Statistical significance was determined using *t*-test

To identify potential biomarkers for pulmonary fibrosis, we analyzed microarray datasets (GSE71351 and GSE134692) from IPF patients. Gene Ontology (GO) analysis revealed a significant increase in the expression of cytokines, chemokines, and growth factors in these datasets. In the GSE71351 dataset, 377 cytokines, chemokines, and growth factors exhibited at least a 1.5-fold increase compared with normal levels, and 140 such genes were identified in the GSE134692 dataset. Forty-nine genes were common to both datasets (Fig. 1a). To investigate their role in pulmonary fibrosis, network analysis was performed, identifying four genes, CSF3, FGF1, IL1β, and CCL11, potentially relevant to the fibrosis process (Supplementary Fig. 1a). Among these genes, CSF3 showed the most significant increase in gene expression in both the GSE dataset (GSE134692 and GSE71351) (Fig. 1b) and in the bleomycin (BLM)-induced IPF mouse model (Supplementary Fig. 1b).

Further analysis of IPF patient-derived fibroblasts (IPDF) demonstrated that knockdown of CSF3, along with FGF1, IL1β, and CCL11, resulted in changes in fibrosis marker expression. CSF3 had the most profound effect on the expression of fibrosis markers (Supplementary Fig. 1c). In addition, CSF3 expression was significantly higher in lung tissue from IPF patients compared to control, as confirmed by immunohistochemical (IHC) staining of pulmonary fibrosis tissue arrays (Fig. 1c). Compared with those of healthy controls, quantitative assessment via immunohistochemistry (IHC) revealed a marked increase in CSF3 expression in IPF lung tissue, which was also reflected in significantly elevated serum levels of CSF3 in IPF patients (Fig. 1d–f).

In the BLM-induced mouse model of pulmonary fibrosis, increased CSF3 expression was also demonstrated in lung tissues via IHC staining (Fig. 1g). Both CSF3 and α-smooth muscle actin (α-SMA) protein levels were significantly upregulated in IPF mouse lungs (Fig. 1h, i), and CSF3 secretion was notably elevated in both lung tissue and blood serum (Fig. 1j, k). Gene set enrichment analysis (GSEA) showed that CSF3 expression was positively correlated with gene signatures related to ECM remodeling and epithelial–mesenchymal transition (EMT), processes known to play crucial roles in fibrosis (Fig. 1l).

Additionally, immunostaining for the CSF3 receptor (CSF3R) revealed elevated expression of CSF3R in both IPF patient samples and BLM-induced IPF mice, consistent with the increased expression of CSF3 (Supplementary Fig. 2a–e). These findings suggest that CSF3 plays a critical role in the fibrotic processes of pulmonary fibrosis in both human and mouse models, making it a promising therapeutic target.

### CSF3 knockout attenuates pulmonary fibrosis progression

**Fig. 2:**
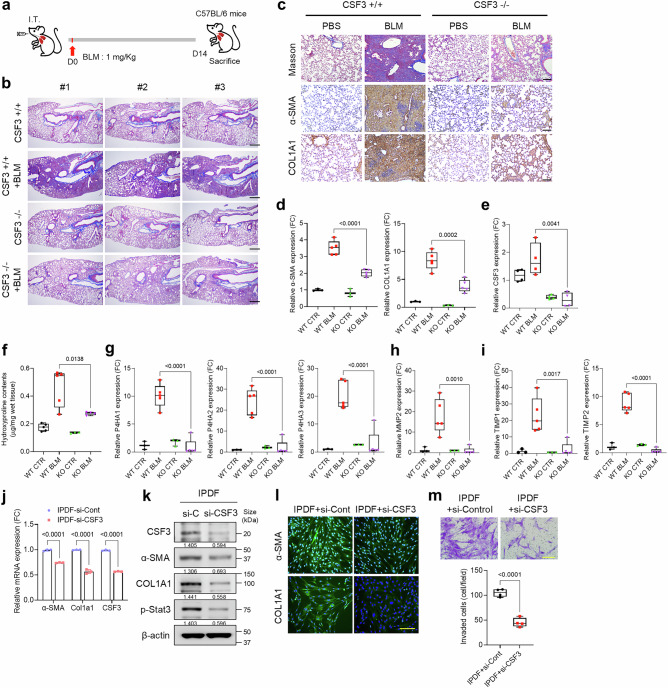
CSF3 knockout attenuates pulmonary fibrosis progression. **a** Schematic illustration of the bleomycin (BLM) intratracheal injection (IT) animal model and the procedure used to induce lung fibrosis in C57BL/6 mice. **b** and **c** Representative Masson’s trichrome staining (**b**, **c**) and immunohistochemistry for α-SMA and COL1A1 (**c**) of lung tissue from CSF3^+/+^ and CSF3^−/−^ mice after BLM or PBS treatment (*n* = 5 per group). Scale bar: 25 μm, Scale bar: 100 μm. **d–i** qRT-PCR analysis of α-SMA, COL1A1, and CSF3 expression (**d**, **e**), hydroxyproline content assay (**f**), qRT-PCR analysis of prolyl hydroxylases (P4HA1, P4HA2, P4HA3) (**g**), matrix metalloproteinase 2 (MMP2) (**h**) and tissue inhibitors of metalloproteinases (TIMP1/2) (**i**) expression in lung tissue from CSF3^+/+^ and CSF3^−/−^ mice after BLM or PBS treatment. **j** qRT-PCR analysis of α-SMA, COL1A1, and CSF3 expression in human lung fibroblasts isolated from idiopathic pulmonary fibrosis patients (IPDF) and transfected with siRNA targeting CSF3 (si-CSF3). **k** Western blot analysis of CSF3, αSMA, COL1A1, and p-STAT3 in IPDF cells transfected with si-CSF3 in IPDF. β-Actin was used as a loading control. **l** Representative immunofluorescence images of α-SMA and COL1A1 in IPDF transfected with si-CSF3. Scale bars: 200 μm. **m** The spontaneous Matrigel-invading capacity of IPDF transfected with si-CSF3 or si-Control. Scale bars: 200 μm. Statistical significance was determined using ANOVA with multiple comparison or *t*-test

To investigate the role of CSF3 in pulmonary fibrosis, we used a CSF3 knockout (KO) mouse model and administered BLM via intratracheal injection (Fig. 2a). Fourteen days post-BLM treatment, lung tissues were collected and analyzed by Masson’s trichrome staining and immunostaining for α-SMA and collagen. Wild-type (WT) mice presented significant fibrotic changes after BLM exposure, while CSF3 KO mice exhibited reduced fibrosis marker expression and severity (Fig. 2b, c). Gene expression analysis confirmed lower levels of α-SMA, COL1A1, and CSF3 in CSF3 KO lungs, indicating reduced fibrosis compared to WT mice (Fig. 2d, e). Further analysis of collagen deposition revealed no increase in hydroxyproline content or hydroxylase activity in CSF3 KO mice, in contrast to the significant increase observed in WT mice following BLM injection (Fig. 2f, g and Supplementary Fig. 3c). Gene expression analysis revealed the upregulation of hydroxylase family members in the IPF patient datasets compared with the control datasets (Supplementary Fig. 3a, b). Additionally, the expression of matrix metalloproteinase 2 (MMP2) and tissue inhibitor of metalloproteinases (TIMP1/2) was elevated in WT mice after BLM treatment, but these genes were not induced in CSF3 KO mice (Fig. 2h, i). In IPDF and BLM-induced IPF mouse lung fibroblasts (BLM-MLF), CSF3 knockdown reduced fibrosis marker expression and phospho-STAT3 levels, similar to the effects observed in CSF3 KO mice (Fig. 2j–l and Supplementary Fig. 4a–c). Furthermore, CSF3 knockdown decreased the invasive capacity of both human and mouse IPF fibroblasts (Fig. 2m and Supplementary Fig. 4d). These findings suggest that CSF3 plays a critical role in pulmonary fibrosis progression, and targeting CSF3 may provide a potential therapeutic approach for IPF.

### CSF3 induces pulmonary fibrosis through the CSF3R/STAT3 signaling axis

**Fig. 3:**
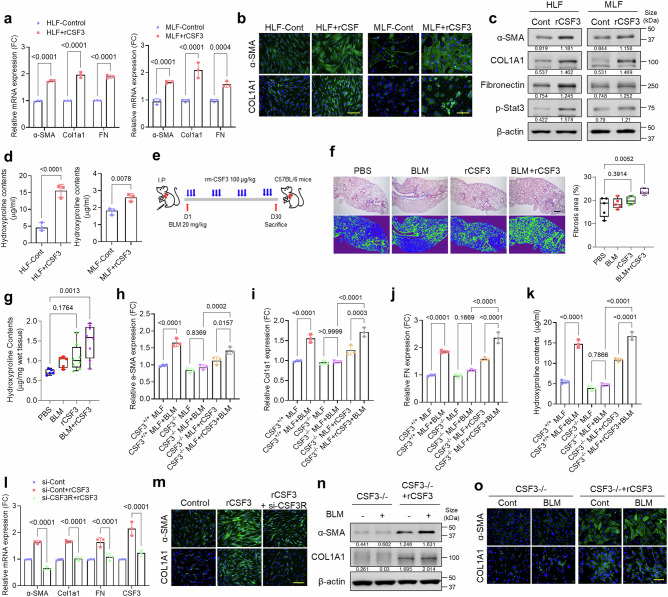
CSF3 induces pulmonary fibrosis through the CSF3R/STAT3 signaling axis. **a** qRT-PCR analysis of fibrosis markers (α-SMA, COL1A1, fibronectin) in human lung fibroblast cell line (HLF, left) and mouse primary lung fibroblast cells (MLF, right) treated with recombinant CSF3 (rCSF3) (200 ng/ml, 24 h). **b** and **c** Representative immunofluorescence images of α-SMA and COL1A1 (**b**), and Western blot analysis of fibrosis markers and STAT3 activation (p-STAT3) (**c**) in HLF and MLF cells treated with rCSF3. β-Actin was used as a loading control. Scale bars: 200 μm. **d** Hydroxyproline content assay in HLF and MLF cells treated with rCSF3. **e** Schematic illustration of the procedure used to induce lung fibrosis in C57BL/6 mice with recombinant mouse CSF3. BLM was administered IP on day 1, followed by IP injections of rCSF3 three times per week. **f** Quantification of the fibrosis area was performed using Orbit software based on Masson’s trichrome staining images. The green area represents the fibrotic region. Scale bar: 500 μm. **g** Hydroxyproline content in mouse lung tissues from groups treated with BLM and rmCSF3 as indicated. **h–k** qRT-PCR analysis of α-SMA (**h**), COL1A1 (**i**) and fibronectin (**j**) expression, and hydroxyproline content (**k**) assay in wild-type (CSF3^+/+^) MLF cells and CSF3 knock-out (CSF3^−/−^) MLF cells treated with BLM and rCSF3. **l** and **m** qRT-PCR analysis of fibrosis markers and CSF3 expression (**l**), and representative immunofluorescence images of α-SMA and COL1A1 (**m**) in HLF cells transfected with si-CSF3R and treated with rCSF3 (200 ng/ml, 24 h). Scale bars: 200 μm. **n** and **o** Western blot analysis of α-SMA and COL1A1 (**n**), and representative immunofluorescence images of α-SMA and COL1A1 (**o**) in CSF3^−/−^ MLF cells treated with BLM and rCSF3. β-Actin was used as a loading control. Scale bars: 200 μm. Statistical significance was determined using ANOVA with multiple comparison or *t*-test

To investigate whether CSF3 contributes to the induction of pulmonary fibrosis, recombinant CSF3 (rCSF3) was administered to human and mouse lung fibroblasts (HLF and MLF). Treatment with rCSF3 increased the expression of fibrosis markers in a concentration-dependent manner (Fig. 3a, b and Supplementary Fig. 5a–d). Western blot analyses confirmed the dose-dependent upregulation of fibrosis markers and phospho-STAT3 (Fig. 3c and Supplementary Fig. 5e). Additionally, rCSF3 treatment resulted in increased hydroxyproline content in both HLF and MLF cells (Fig. 3d and Supplementary Fig. 5f). To further validate the role of CSF3 in pulmonary fibrosis, a C57BL/6 mouse model was utilized. The mice were intraperitoneally injected with BLM and rCSF3 (Fig. 3e). The application of Masson’s trichrome staining and subsequent image analysis indicated that each individual treatment group exhibited slight induction of collagen deposition across the lung tissue, accompanied by the presence of fibrotic areas. In contrast, the combination treatment group demonstrated significant induction of collagen deposition (Fig. 3f). Interestingly, the hydroxyproline content was markedly elevated in both the CSF3 and combination treatment groups (Fig. 3g). Furthermore, in CSF3^−/−^ primary MLFs, rCSF3 treatment restored the expression of fibrosis markers and hydroxyproline content (Fig. 3h–k). In addition, knockdown of CSF3R gene expression blocked the induction of fibrosis markers by rCSF3 (Fig. 3l, m). CSF3^−/−^ MLFs exhibited inhibited fibrosis marker expression in response to BLM, but rCSF3 restored the expression of these markers (Fig. 3n, o). Since STAT3 is the principal downstream mediator of CSF3R signaling, treatment with a STAT3 inhibitor suppressed the rCSF3-induced increase in fibrosis markers (Supplementary Fig. 5g, h). These findings support that CSF3 plays a pivotal role in the induction of pulmonary fibrosis through the activation of the CSF3R/STAT3 signaling axis.

### Evaluation of CSF3-neutralizing antibody FB-101 in pulmonary fibrosis models

**Fig. 4:**
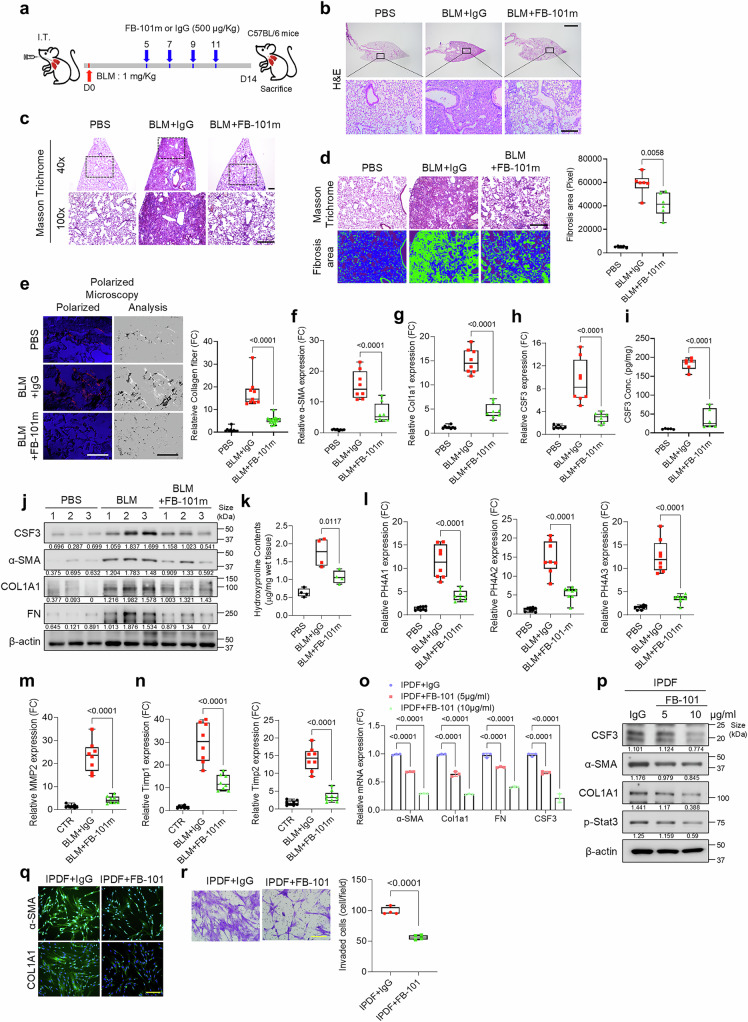
Evaluation of CSF3-neutralizing antibody FB-101 in pulmonary fibrosis models. **a** Schematic representation of the BLM IT injection animal model, the generation of lung fibrosis, and the overall therapeutic procedure in C57BL/6 mice. The mice CSF3 neutralizing antibody (FB-101m) and IgG were administered I.P. **b** and **c** Representative H&E (**b**) and Masson’s trichrome staining (**c**) images of lung tissue from each mouse group (*n* = 5 per group). Scale bar: 2 mm and 250 μm. **d** Quantification of the fibrotic area from the Masson’s trichrome staining images using Orbit software. Scale bar: 250 μm. **e** Picrosirius red-stained therapeutic mouse model samples were analyzed by polarized microscopy. Collagen fibers (left, polarized light) and their subsequent analysis using ImageJ (right, analysis) provide a quantitative assessment of the fibers. Scale bar: 100 μm. **f–j** qRT-PCR analysis of α-SMA (**f**), COL1A1 (**g**), and CSF3 (**h**) expression, ELISA quantification of CSF3 levels (**i**), and Western blot analysis of CSF3, α-SMA, COL1A1, and fibronectin (FN) (**j**) in lung tissue from each mouse group. β-Actin was used as a loading control. **k–n**, Hydroxyproline content assay (**k**), qRT-PCR analysis of prolyl hydroxylase family (P4HA1, P4HA2, P4HA3) (**l**), matrix metalloproteinase 2 (MMP2) (**m**), and tissue inhibitors of metalloproteinases (TIMP1/2) (**n**) expression in lung tissue from each mouse group. **o–q** qRT-PCR analysis of α-SMA, COL1A1, FN, and CSF3 expression (**o**), Western blot analysis of CSF3, α-SMA, COL1A1, and phospho-STAT3 (p-STAT3) (**p**), and representative immunofluorescence images of α-SMA and COL1A1 (**q**) in IPDF treated with the human CSF3 neutralizing antibody (FB-101). β-Actin was used as a loading control. Scale bar: 200 μm. **r** The spontaneous Matrigel-invading capacity of IPDF treated with FB-101 or IgG. Scale bar: 200 μm. Statistical significance was determined using ANOVA with multiple comparisons or a *t*-test

To evaluate the therapeutic potential of neutralizing CSF3 in pulmonary fibrosis, we developed a CSF3-neutralizing antibody, FB-101, and assessed its efficacy in two distinct BLM-induced IPF mouse models. The reversal therapeutic effect was achieved by IP injection of FB-101 starting 5 days after BLM injection (Fig. 4a), and the preventive protection effect was achieved by IP injection of FB-101 starting 1 day after BLM treatment (Supplementary Fig. 6a). The antibody was administered post-BLM treatment, and the histological outcomes were analyzed. FB-101-treated mice showed significant reductions in BLM-induced fibrotic changes, including alveolar wall destruction, collagen deposition, and fibrosis area expansion (Fig. 4b–d and Supplementary Fig. 6b, c). Sirius red staining of collagen fibers under polarized light microscopy further confirmed the reduced collagen deposition in FB-101-treated mice (Fig. 4e and Supplementary Fig. 6d). FB-101 treatment significantly suppressed the increase in BLM-induced fibrosis markers (Fig. 4f–j), and the hydroxyproline content and hydroxylase expression were notably reduced (Fig. 4k, l and Supplementary Fig. 6e). Furthermore, the levels of MMP2 and TIMP1/2 induced by BLM were reduced in FB-101-treated mice (Fig. 4m, n).

In vitro experiments with IPDF and BLM-MLF confirmed that FB-101 inhibited fibrosis marker expression and reduced CSF3 levels (Fig. 4o–q and Supplementary Fig. 7a–c). Additionally, FB-101 blocked the CSF3R/STAT3 signaling pathway (Fig. 4p), with the inhibitory effect persisting for up to 72 h post-treatment (Supplementary Fig. 8a). Conversely, treatment with BLM and CSF3R overexpression led to the induction of CSF3R/STAT3 binding in normal human bronchial epithelial cells (Supplementary Fig. 7d). Furthermore, treatment of FB-101 antibody in IPDF decreased binding of phospho-STAT3 to the CSF3 and TGF-β1 promoters. (Supplementary Fig. 7e). The suppression of fibrosis markers and CSF3 expression was observed, with maximal efficacy at concentrations of 10 µg/ml or higher (Supplementary Fig. 8b). FB-101 treatment also reduced cell invasion in both IPDF and BLM-MLF (Fig. 4r and Supplementary Fig. 7f).

An additional intraperitoneal mouse model induced by BLM confirmed that FB-101 treatment significantly suppressed fibrotic changes in lung tissue, inhibited fibrosis marker expression, and reduced the hydroxyproline content (Supplementary Fig. 9a–f). Furthermore, FB-101 was observed to reduce the expression of EMT markers, ECM proteins, and TIMP1/2 (Supplementary Fig. 9g–j). These findings indicate that FB-101 may have therapeutic potential in mitigating pulmonary fibrosis through CSF3 neutralization.

### CSF3 acts as an upstream regulator of TGF-β in pulmonary fibrosis

We demonstrated that the loss of CSF3 function, achieved through neutralizing antibody treatment or gene expression depletion, can prevent and restore the development of pulmonary fibrosis in mouse models. Given the crucial role of TGF-β1 in pulmonary fibrosis, including its involvement in EMT, fibroblast-to-myofibroblast transition (FMT), ECM deposition, and myofibroblast activation, we aimed to investigate the relationship between CSF3 and TGF-β1 in IPF pathogenesis. GSEA of high versus low CSF3 expression groups in the IPF cohort revealed a positive correlation between TGF-β1 and CSF3 expression (Supplementary Fig. 10a).

**Fig. 5:**
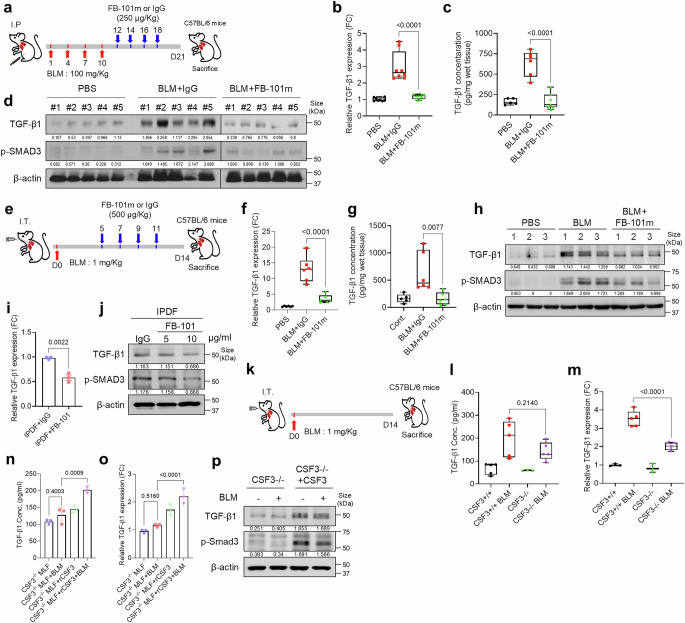
CSF3 acts as an upstream regulator of TGF-β in pulmonary fibrosis. **a** Schematic illustration of the BLM I.P injection mouse model, the generation of lung fibrosis, and the overall therapeutic procedure in C57BL/6 mice. The anti-mouse CSF3 antibody (FB-101m) and IgG were administered via intraperitoneal injection. **b–d** qRT-PCR (**b**), ELISA quantification (**c**), and Western blot (**d**) analysis of TGF-β1 level in lung tissue from each BLM IP injection mouse group. β-Actin was used as a loading control. **e** Schematic illustration of the BLM IT injection mouse model, the generation of lung fibrosis, and the overall therapeutic procedure in C57BL/6 mice. FB-101m and IgG were administered intraperitoneally. **f–h** qRT-PCR analysis of TGF-β1 expression (**f**), ELISA quantification of TGF-β1 levels (**g**), and Western blot of TGF-β1 and phospho-SMAD3 levels (**h**) in lung tissue from each BLM I.T. mouse group. β-Actin was used as a loading control. **I** and **j** qRT-PCR analysis of TGF-β1 expression (**i**) and Western blot analysis of TGF-β1 and phospho-SMAD3 levels (**j**) in IPDF treated with the neutralizing antibody FB-101. β-Actin was used as a loading control. **k** Schematic illustration of the BLM IT animal model, the generation of lung fibrosis, and the overall therapeutic procedure in C57BL/6 CSF3^+/+^ and CSF3^−/−^ mice. **l** and **m** ELISA quantification of TGF-β1 levels (**l**) and qRT-PCR analysis of TGF-β1 expression (**m**) in lung tissue from each C57BL/6 CSF3^+/+^ and CSF3^−/−^ mouse group. **n–p**, ELISA quantification of TGF-β1 levels (**n**), qRT-PCR analysis of TGF-β1 expression (**o**), and Western blot analysis of TGF-β1 and phospho-SMAD3 levels (**p**) in CSF3 knock-out (CSF3^−/−^) MLF cells. The cells were treated with BLM and CSF3 as indicated. β-Actin was used as a loading control. Statistical significance was determined using ANOVA with multiple comparisons or *t*-test

To assess the role of CSF3 in regulating TGF-β1 expression in IPF, we utilized both intraperitoneal and intratracheal BLM-induced mouse models, treating the mice with FB-101 (Fig. 5a, e). FB-101 treatment significantly reduced the expression of TGF-β1 mRNA (Fig. 5b, f), protein, and level of phospho-SMAD3 (Fig. 5d, h) in IPF mouse lung tissue compared to the control group. FB-101 selectively targets excessive TGF-β induced by pulmonary fibrosis, without altering the basal levels of TGF-β1, which are crucial for homeostasis. Furthermore, the treatment effectively diminished the secretion of TGF-β1 (Fig. 5c, g). Furthermore, the treatment of rCSF3 in HLF resulted in a dose-dependent increase in the expression of TGF-β1 (Supplementary Fig. 10b–d) and other fibrosis-associated molecules, including IL-11 and CTGF, which are downstream of TGF-β1 (Supplementary Fig. 10e). Notably, in IPDF, FB-101 treatment resulted in a significant reduction in TGF-β1 expression and inhibition of the downstream signaling mediator SMAD3 activation (Fig. 5i, j and Supplementary Fig. 10f).

To confirm these findings, we used a CSF3 KO model (Fig. 5k). After 14 days of BLM administration, lung tissue from CSF3 KO mice showed a significant reduction of TGF-β1 expression (Fig. 5l) and secretion (Fig. 5m) compared to WT controls. Moreover, rCSF3 administration restored TGF-β/SMAD3 signaling in CSF3^−/−^ MLFs (Fig. 5n–p). Additionally, CSF3 neutralization in IPDF-mimicking BLM-MLF cultures inhibited both TGF-β1 expression and TGF-β/SMAD3 signaling (Supplementary Fig. 10g, h). Knockdown of CSF3 in BLM-MLF did not induce TGF-β1 expression (Supplementary Fig. 10i), further supporting the critical role of CSF3 in TGF-β1 regulation. These findings indicate the essential role of CSF3 in regulating TGF-β1 expression and TGF-β/SMAD3 signaling in pulmonary fibrosis.

To further investigate the role of CSF3 in TGF-β1 signaling in IPF pathogenesis, we silenced TGF-β receptor II (TGF-βRII) in HLF treated with rCSF3. As expected, the induction of fibrosis marker expression by CSF3 was significantly reduced when TGF-βRII was knocked down (Supplementary Fig. 11a–c). To explore the involvement of CSF3 in TGF-β1-mediated fibrosis, we treated HLF with recombinant TGF-β1 and observed an unexpected increase in CSF3 expression (Supplementary Fig. 11d). The increase in fibrosis markers induced by TGF-β1 were attenuated following CSF3 knockdown (Supplementary Fig. 11e), and the downstream mediator IL-11 was diminished (Supplementary Fig. 11f). Additionally, treatment with FB-101, significantly reduced TGF-β1-induced fibrosis markers (Supplementary Fig. 11g, h) and prolyl-hydroxylases (Supplementary Fig. 11I), and reversed the expression of TIMP1/2 (Supplementary Fig. 11j). The treatment of recombinant TGF-β1 resulted in an enhancement of phospho-SMAD2/3 binding to the CSF3 and TGF-β1 promoters. Conversely, treatment with FB-101 led to a reduction in phospho-SMAD2/3 binding to these promoters (Supplementary Fig. 11k). These findings suggest a positive feedback loop between CSF3 and TGF-β1, contributing to IPF progression.

### CSF3 neutralization restores alveolar type 2 cells and modulates immune cell recruitment in pulmonary fibrosis

**Fig. 6:**
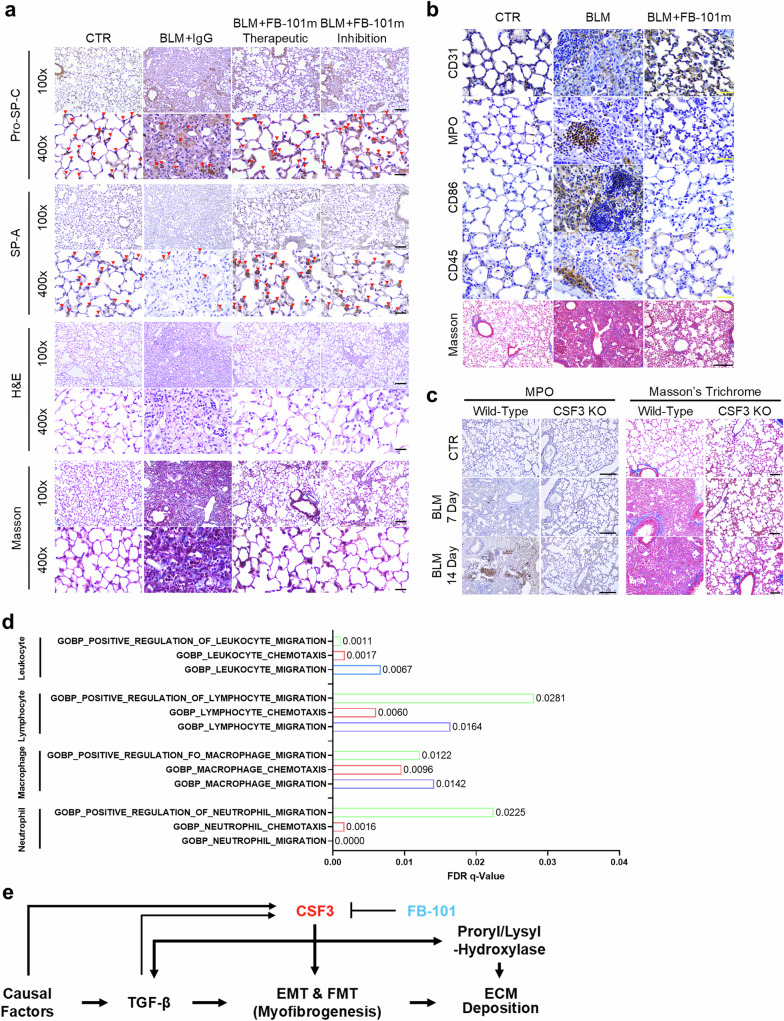
CSF3 neutralization restores alveolar type 2 cells and modulates immune cell recruitment in pulmonary fibrosis. **a** H&E, Masson’s trichrome staining, and immunostaining (pro-SP-C (pro-Surfactant protein C), and SP-A (Surfactant protein A), alveolar type 2 cell marker) of lung tissue from IT therapeutic and inhibition BLM model. The mouse CSF3 neutralizing antibody (FB-101m) was administered IP ×100; Scale bar 100 μm. ×400; Scale bar: 25 μm. **b** Masson’s trichrome staining and immunostaining (CD31; endothelial, MPO; neutrophil, CD86; macrophage, and CD45; pan-leukocyte) of lung tissue from C57BL/6 N mice intubated with BLM or PBS. The mouse CSF3 neutralizing antibody (FB-101m) was administered intraperitoneally. Masson’s trichrome Scale bar: 250 μm. Immunostaining Scale bar: 50 μm. **c** Masson’s trichrome staining and immunostaining (MPO; neutrophile) of lung tissue from CSF3^+/+^ and CSF3^−/−^ mice intubated with BLM or PBS. Sacrifice was performed on each date, 7 days and 14 days after BLM treatment. (CTR *n* = 3; BLM 7, 14 days *n* = 5). Scale bar: 250 μm. **d** GSEA FDR values for features related to the recruitment of neutrophils, macrophages, lymphocytes, and leukocytes by high/low CSF3 expression. **e** Schematic summary of the mechanism of CSF3-induced fibrosis and proposed therapeutic strategy

In response to various lung injuries, alveolar type 2 (AT2) cells undergo replication and expansion to facilitate tissue repair. However, in the context of pulmonary fibrosis, a pathological condition characterized by excessive scarring, the population of AT2 cells, which are essential for repair, is depleted. In this study, we observed that the depletion of AT2 cells in the lungs during pulmonary fibrosis was restored upon treatment with FB-101 in two distinct BLM-induced IPF mouse models (therapeutic and inhibition) (Fig. 6a). This restoration of AT2 cell numbers coincided with the resolution of pulmonary fibrosis, restoring collagen, pro-surfactant protein C, and surfactant protein A-positive cells (Fig. 6a), suggesting a potential therapeutic role for CSF3 neutralization-based interventions in restoring cellular homeostasis and promoting fibrosis resolution. To investigate the morphology of blood vessels and the recruitment of neutrophils and macrophages in the lungs of mice with BLM-IPF, we performed immunohistochemical staining for CD31 (endothelial cell marker), MPO (myeloperoxidase, a neutrophil marker), CD86 (macrophage marker), and CD45 (pan-leukocyte marker). In the BLM-IPF mouse model, the morphology of blood vessels appeared disorganized, and there was increased recruitment of neutrophils and macrophages. However, following treatment with FB-101, the blood vessels were restored to normal morphology, and the recruitment of neutrophils, macrophages, and leukocytes was significantly reduced, and AT2 cell populations were restored. (Fig. 6b). In A549 (AT2; alveolar cell type II phenotype lung cancer cell), TGF-β1 treatment induced myofibroblast marker and CSF3 expression, but surfactant protein expression was reduced, which was restored by FB-101 treatment (Supplementary Fig. 12a). Additionally, as anticipated, the elevated neutrophil recruitment observed in the BLM-IPF mice was notably attenuated in the CSF3 KO mice (Fig. 6c). In support of this result, GSEA data analysis also confirmed that when CSF3 is high, a set of genes related to the recruitment of neutrophils, macrophages, lymphocytes, and leukocytes showed a positive correlation (Fig. 6d and Supplementary Fig. 12b) These results indicate the potential for CSF3 neutralization-based interventions to play a role in restoring cellular homeostasis and promoting fibrosis resolution.

Collectively, these findings provide compelling evidence that CSF3 plays a crucial role in the progression of pulmonary fibrosis. Our data demonstrate that CSF3 promotes myofibroblast activation and ECM deposition in lung fibroblasts. Mechanistically, CSF3 functions as an upstream regulator of TGF-β, promoting its expression and signaling. Furthermore, CSF3 acts as a key effector in the progression of pulmonary fibrosis induced by TGF-β. The positive feedback loop between CSF3 and TGF-β appears to constitute a central pathway that regulates both the initiation and progression of pulmonary fibrosis. Furthermore, neutralizing CSF3 can reduce fibrosis by inhibiting TGF-β signaling, deactivating myofibroblasts, and restoring the ECM and cellular homeostasis. These findings position CSF3 as a promising therapeutic target for IPF, with the potential to improve patient outcomes by halting disease progression (Fig. 6e).

## Discussion

IPF is a progressive and fatal lung disease with no definitive cure. Currently, FDA-approved antifibrotic drugs primarily serve as palliative treatments, slowing disease progression but failing to halt or reverse the underlying pathological processes of IPF.^2,27,28^ The rising global incidence and mortality rate of IPF,^29,30^ which surpasses that of many cancers, underscores the urgent need for more effective therapeutic strategies. This critical treatment gap highlights the importance of identifying novel molecular targets, which could provide new avenues for intervention and lead to the development of more effective therapies.

In the present study, we identify CSF3 as a key cytokine in the progression of IPF. Our findings show that CSF3 expression is significantly elevated in lung tissues from IPF patients compared with healthy controls. This increase in CSF3 levels is associated with fibrotic mediators and processes such as ECM remodeling, EMT and FMT, which are central to the pathogenesis of fibrosis.^31–33^ Immunohistochemical analysis further confirmed the marked elevation of CSF3 in the lung tissue of IPF patients. Importantly, the expression of CSF3 was correlated with genes involved in ECM remodeling and fibrogenesis, suggesting that CSF3 plays a pivotal role in the development and progression of pulmonary fibrosis.

One of the intriguing findings of this study is the elevation of CSF3 in IPF tissues, despite the absence of significant inflammation after disease onset. Traditionally, CSF3 is known to regulate neutrophil survival, proliferation, and recruitment, with neutrophils playing a central role in acute inflammation.^34,35^ However, in IPF, inflammation is minimal, yet neutrophils persist in fibrotic tissue.^36^ This paradox suggests that CSF3 might have a more complex role in fibrosis than previously understood. By promoting neutrophil recruitment, CSF3 may facilitate the sustained presence of neutrophils in fibrotic tissue, where they contribute to tissue remodeling and the perpetuation of fibrosis through mechanisms such as fibroblast activation, differentiation, and ECM deposition.^35,37,38^ Additionally, neutrophils release proteases such as neutrophil elastase and MMPs, which degrade ECM components and activate latent TGF-β, further exacerbating fibrosis.^39–41^ These findings suggest that CSF3 plays a critical role in neutrophil dynamics within fibrotic tissue, and emphasize the need to explore its direct effects on fibrosis independent of neutrophils.

However, our findings demonstrate that recombinant human CSF3 administration significantly enhances fibrogenesis in human lung fibroblasts, promoting their differentiation into myofibroblasts and driving increased expression of key ECM components. In contrast, knockout of the CSF3 gene confers protection against bleomycin-induced lung fibrosis, suggesting that CSF3 is integral to the fibrotic response. Furthermore, the administration of CSF3-neutralizing antibodies resulted in a notable reversal of pulmonary fibrosis in IPF mouse models, supporting the hypothesis that CSF3 is a central mediator in the development and maintenance of fibrosis. This therapeutic effect was associated with a reduction in TGF-β expression and signaling, deactivation of myofibroblasts, and restoration of collagen homeostasis. These findings provide direct evidence linking CSF3 to the progression of pulmonary fibrosis, thereby supplementing the currently limited understanding of CSF3’s effects on fibroblasts and the fibrotic process. In addition to extending previous research that primarily focused on the role of CSF3 in neutrophil biology, these results underscore the potential of targeting CSF3 as a viable therapeutic strategy for IPF.

Neutrophils play a multifaceted role in IPF. While they are essential for the early inflammatory response to lung injury, their continued presence in fibrotic tissue has been linked to disease progression.^42^ CSF3, by modulating neutrophil recruitment and survival, may facilitate the persistence of neutrophils in fibrotic areas,^35,38,43^ where they contribute to tissue damage and fibrosis through the release of proteases and the activation of TGF-β.^18,24,44,45^ Our results suggest that CSF3 plays a critical role in the recruitment of neutrophils to fibrotic sites and that its direct effects on fibroblast differentiation into myofibroblasts further implicate CSF3 as a central player in IPF pathogenesis. This highlights the dual role of CSF3 in both neutrophil dynamics and fibroblast activation, making it an important target for therapeutic intervention.

The relationship between CSF3 and TGF-β is of particular interest. TGF-β is a principal driver of fibrosis and is capable of inducing fibroblast activation, myofibroblast differentiation, and ECM production.^46–48^ Nevertheless, direct targeting of TGF-β has proven to be a significant challenge due to its pleiotropic effects and its essential roles in various biological processes.^48,49^ For example, patients treated with fresolimumab, a broad-spectrum TGF-β inhibitor, experienced severe side effects, including infections and autoimmune-like symptoms, due to interference with TGF-β‘s role in maintaining immune balance and normal physiological functions.^50,51^ Notably, our study suggests that inhibition of CSF3 selectively targets the excessive TGF-β induced by pulmonary fibrosis, without altering the basal levels of TGF-β that are crucial for homeostasis. Furthermore, we demonstrate that CSF3 functions as an upstream regulator of TGF-β, promoting its expression and signaling through a positive feedback loop that exacerbates fibrosis. By targeting CSF3, we may be able to modulate TGF-β activity in a more specific and controlled manner, potentially reducing the adverse effects associated with direct TGF-β inhibition.

Additionally, our findings indicate that CSF3 influences the expression of prolyl 4-hydroxylase alpha (P4HA), a pivotal enzyme implicated in collagen synthesis and fibrosis.^52^ Both P4HA expression and hydroxyproline levels were elevated in IPF patient cohorts and bleomycin-induced mouse models of IPF.^53,54^ Treatment with CSF3 further increased P4HA expression in human lung fibroblasts, while CSF3 neutralization reduced P4HA levels, indicating that CSF3 plays a crucial role in collagen deposition and ECM remodeling. Moreover, we demonstrate that treatment with FB-101 led to a significant restoration of alveolar type 2 (AT2) cell populations, improved blood vessel morphology, and a reduction in the recruitment of neutrophils and macrophages. Collectively, these results suggest that CSF3 neutralization holds promise as an intervention strategy for restoring cellular homeostasis, enhancing tissue repair, and promoting the resolution of fibrosis. The therapeutic potential of targeting CSF3 for modulating immune cell recruitment and ECM remodeling indicates its relevance in treating fibrotic diseases such as IPF.

Despite these promising results, several limitations should be recognized. While the bleomycin-induced mouse model is widely used, it may not fully capture the chronic progression and complex etiology of human IPF. To complement this limitation, recent clinical studies have implicated CSF3 in the pathogenesis of IPF. Elevated CSF3 levels have been detected in the bronchoalveolar lavage fluid (BALF) of IPF patients, with significant correlations to impaired lung function and poor prognosis.^18^ Moreover, CSF3 has been associated with acute exacerbations of IPF, where it increases alveolar epithelial barrier permeability and promotes inflammatory cell infiltration, thereby exacerbating lung injury. These observations suggest that CSF3 contributes not only to chronic fibrosis but also to acute exacerbation events, reinforcing its therapeutic potential in both aspects of IPF pathology.

In conclusion, our study identifies CSF3 as a critical cytokine involved in the initiation, progression, and maintenance of pulmonary fibrosis. By acting as an upstream regulator of TGF-β, CSF3 enhances key fibrotic pathways and promotes the pathological processes of IPF. Targeting CSF3 with neutralizing antibodies represents a promising therapeutic strategy to restore fibrosis, selectively suppress excessive TGF-β signaling while preserving normal homeostatic functions. Given the limitations of current therapies, CSF3-targeted treatments offer a novel approach to address the unmet need for more effective therapies for IPF. Our findings provide a strong rationale for further clinical investigations into CSF3 inhibition as a potential treatment for IPF and other fibrotic diseases. However, although antibody-based therapies such as CSF3-neutralizing antibodies exhibit high target specificity, the possibility of off-target effects cannot be entirely excluded. Therefore, comprehensive evaluation through toxicological and safety studies will be essential to ensure clinical applicability.

## Materials and Methods

### Experimental animals and bleomycin (BLM)-induced pulmonary fibrosis model

The Animal study was approved by the Hanyang University Institutional Animal Care and Use Committee (IACUC) (IACUC No. 202270107 A). Seven-week-old C57BL/6N female mice (weighing ~20 g) were purchased from Orient Bio, and pulmonary fibrosis was induced via intraperitoneal or intratracheal (IT) bleomycin (BLM) injection. Briefly, BLM was administered at 1 mg/kg once and 100 mg/kg four times, respectively, to establish IT and intraperitoneal (IP) pulmonary fibrosis models. To determine the effect of CSF3 inhibition on preventing pulmonary fibrosis, randomly divided groups of mice received four IP injections of PBS, control IgG, or anti-CSF3 antibody (250 μg/kg) at intervals of 2 days (IT model) or 3 days (IP model), the day after BLM administration. Mice were monitored daily and sacrificed 14 days (IT model) or 21 days (IP model) after BLM administration to collect fresh lungs. The left lung was used for histological analysis, and the right lung was used for molecular biochemical analysis, such as qRT-PCR, Western blotting, and hydroxyproline assays.

The therapeutic effect of neutralizing CSF3 antibodies (FB-101) on pulmonary fibrosis was assessed by delayed administration of antibodies to BLM-injected mice. Antibodies were administered intraperitoneally at 500 μg/kg for the IT model and 250 μg/kg for the IP model four times every other day.

The CSF3-deficient mice were generated at Macrogen Inc. using the CRISPR/Cas9 system in a C57BL/6 background. In a comparative study of BLM-induced pulmonary fibrosis in CSF3-deficient and wild-type mice, BLM was administered at 1 mg/kg once for IT injection and 100 mg/kg four times for IP injection.

For the CSF3-induced fibrosis model, low doses of BLM were treated first to induce minimal inflammatory responses, and then recombinant CSF3 was used. Female mice (7-week-old) were given a BLM solution (20 mg/kg, administered intraperitoneally on day 1). Mice were sacrificed on day 30. The 8-week-old female mice were injected intraperitoneally with recombinant mouse CSF3 protein at a concentration of 100 μg/kg every other day for 4 weeks (3 times per week) starting on day 1 to induce pulmonary fibrosis in mice. Mice were then sacrificed on day 30.

### Primary lung fibroblast isolation

Primary mouse lung fibroblasts (MLF) were isolated from whole lungs of wild-type C57BL/6 treated with/without BLM and CSF3 KO (CSF3^−/−^) mice as described previously.^55^ Fresh lungs from neonatal mouse pups or 10-week-old mice with and without BLM administration were perfused and washed at least three times with phosphate-buffered saline (PBS). Lung tissues were cut into ~1 mm pieces using a sterile scalpel, and the tissue fragments were transferred into a 60 mm culture dish. The lung tissue fragments were cultured in DMEM supplemented with 10% FBS and 1% antibiotic/antimycotic solution. Once the growing cells became confluent, they were subcultured by trypsinization. In this study, primary lung fibroblasts passaged 4–6 times were used for experiments.

### Measurement of hydroxyproline content and siRNA knockdown

Hydroxyproline content in mouse lung tissue or primary lung fibroblasts was quantified using a hydroxyproline assay kit (Chondrex, Inc., #6017) using 10 mg of mouse tissues according to the manufacturer’s instructions. Transfection was carried out using Lipofectamine 2000 (Invitrogen) for small interfering RNA (siRNA) according to the manufacturer’s instructions. The TGFβRII or CSF3R siRNA was purchased from Santa Cruz (Cat. No. sc-36657 for TGFβRII) or Genolution, Inc. for CSF3R. The corresponding target sequences are 5’-GGUGUCGUACCGCGUUCUAUU-3’ for CSF3 (i), and 5’-GUUUGACUCCCGAACAUCAUU-3’ for CSF3 (ii), respectively.

### Immunohistochemistry (IHC) and human tissue array (TMA)

In this study, immunohistochemical analysis was performed with the left lung lobe of mice as previously described. In brief, 4 μm-thick sections from formalin-fixed paraffin-embedded lung tissue blocks were deparaffinized with xylene, dipped in 100%, 95%, 80% and 70% ethanol for hydration and washed with tap water for 10 min. Heat-induced epitope retrieval (HIER) was performed using citrate buffer, pH 6.0 (Sigma, #C9999). After blocking with PBS containing 1% bovine serum albumin (BSA), sections were incubated with primary antibodies at 4 °C overnight. After washing with PBST (10% Tween 20 in PBS), the sections were washed with PBST (10% Tween 20 in PBS) and then treated with biotinylated pan-specific secondary antibody (Vector Laboratories, 1:200) for 1 h. After incubation with ABC solution (Vector Laboratories, #PK-6100), the signal visualization was performed with 3,3′-diaminobenzidine (Vector Laboratories, #SK-4100). Counterstaining was performed with hematoxylin for 3 min. Sections were mounted, and images were captured using an IX71 microscope (Olympus). Human pulmonary interstitial fibrosis tissue array samples were purchased from US Biomax Inc. (#LC561a), and IHC was performed using anti-CSF3 or CSF3R antibody according to the IHC analysis protocol. IHC scores were obtained using Image J with the algorithm of the IHC profiler.^56^ Analysis of stained slides was performed using two different methods: counting target protein-positive cells and assessing the staining intensity of target protein expression.

### Gene Expression Omnibus (GEO) and Gene Set Enrichment Analysis (GSEA)

Gene Set Enrichment Analysis (GSEA) was performed to identify differentially expressed genes (DEGs) in patients with idiopathic pulmonary fibrosis (IPF). For this purpose, publicly available RNA-seq and microarray gene expression profiling datasets were obtained from the Gene Expression Omnibus (GEO) and analyzed using GSEA software (version 4.3.3). The datasets from both healthy individuals and IPF patients are publicly accessible via GEO (accession numbers: GSE134692, GSE71351, and GSE10667). Among these, GSE134692, GSE71351, and GSE10667 were used for IPF-specific target identification and gene expression analysis, while GSE10667 was specifically used for GSEA.

### Histological staining

Paraffin-embedded mouse lung samples were cut to obtain 4-μm-thick slices. Sections were deparaffinized and rehydrated for histological staining. For Hematoxylin–Eosin (H&E) staining, lung sections were sequentially subjected to hematoxylin (YD diagnostics, #S2-5), differentiation in 70% ethanol containing 1% HCl, and alcoholic Eosin (BBC Biochemical, # MA0101015). Masson’s trichrome staining of lung sections was conducted according to the manufacturer’s instruction kit (Bio Quochem, #KH07007). Picrosirius red staining was carried out using the Picro Sirius Red Stain Kit (Abcam, #ab150681) according to the manufacturer’s instructions. Collagen fiber was evaluated using a BX50 microscope (Olympus) with a polarizing filter as previously described. Micrographs were digitized into 8-bit grayscale images using ImageJ software. Collagen fiber areas were then quantified in black and white by adjusting the threshold without damaging the original image.

### Generation of fully human or mouse monoclonal antibodies against CSF3 (FB-101, FB-101m)

FB-101 is a fully human monoclonal antibody that specifically binds to human CSF3, developed by Y-Biologics using a human antibody library and phage display technology. The specific binding affinity to human CSF3 was verified through ELISA and SPR, and the binding site was identified through epitope mapping analysis. FB-101m is a mouse monoclonal antibody that specifically binds to mouse CSF3, developed by AbClon using hybridoma technology.

### Fibroblast-to-myofibroblast transition (FMT) assay

Cells (MRC5, AHLF) are seeded in a 60 mm plate at a density of around 3 × 10^5^ (MRC5) or 2.5 × 10^5^ (IPIF, AHLF) cells. Following a 16-h incubation period, the medium is replaced with FBS-free medium and, two hours later, treated with the indicated concentrations of antibodies. After one hour, cells are treated with recombinant TGF-β1 protein (2 ng/ml), and cells are harvested for analysis 24 h later.

### Statistical analysis

All statistical analyses are evaluated by an unpaired Student’s *t*-test (two-tailed) for comparisons between two groups or using a one-way ANOVA followed by Tukey’s multiple comparison procedure for comparisons between more than two groups. Statistical *p*-values for each comparison are specified above the black lines in each figure.

## Supplementary information

Quality check Supplementary Figure 1-12 - Clean version

Western blot Raw data - 1

Western blot Raw data - 2

Western blot Raw data - 3

Western blot Raw data - 4

## Data Availability

The dataset used in this manuscript is publicly available in the Gene Expression Omnibus (GEO) database (accession numbers: GSE134692, GSE71351, and GSE10667). The JASPAR, UCSC Genome Browser, gene set enrichment analysis (GSEA), Image J, and Cytoscape software are currently available on their each websites (https://jaspar.elixir.no; https://genome.ucsc.edu/index.html; https://www.gsea-msigdb.org/gsea/index.jsp; https://imagej.net/ij/; https://cytoscape.org/). GraphPad Prism software (version 10.0; GraphPad Software, San Diego, CA, USA) was used for data presentation and analysis. The software was purchased from the official website (https://www.graphpad.com/).

## References

[1] Martinez, F. J. et al. Idiopathic pulmonary fibrosis. *Nat. Rev. Dis. Prim.* **3**, 1–19 (2017). 10.1038/nrdp.2017.74 29052582

[2] Chianese, M. et al. Pirfenidone and nintedanib in pulmonary fibrosis: lights and shadows. *Pharmaceuticals* **17**, 709 (2024). 38931376 10.3390/ph17060709 PMC11206515

[3] Amati, F. et al. Efficacy of pirfenidone and nintedanib in interstitial lung diseases other than idiopathic pulmonary fibrosis: a systematic review. *Int. J. Mol. Sci.* **24**, 7849 (2023). 37175556 10.3390/ijms24097849 PMC10178294

[4] Finnerty, J. P., Ponnuswamy, A., Dutta, P., Abdelaziz, A. & Kamil, H. Efficacy of antifibrotic drugs, nintedanib and pirfenidone, in treatment of progressive pulmonary fibrosis in both idiopathic pulmonary fibrosis (IPF) and non-IPF: a systematic review and meta-analysis. *BMC Pulm. Med.* **21**, 411 (2021). 34895203 10.1186/s12890-021-01783-1 PMC8666028

[5] Glass, D. S. et al. Idiopathic pulmonary fibrosis: current and future treatment. *Clin. Respir. J.* **16**, 84–96 (2022). 35001525 10.1111/crj.13466 PMC9060042

[6] Fujimoto, H., Kobayashi, T. & Azuma, A. Idiopathic pulmonary fibrosis: treatment and prognosis. *Clin. Med. Insights Circ. Respir. Pulm. Med.* **9**, CCRPM. S23321 (2015). 10.4137/CCRPM.S23321 PMC5147432 27980445

[7] Shenderov, K., Collins, S. L., Powell, J. D. & Horton, M. R. Immune dysregulation as a driver of idiopathic pulmonary fibrosis. *J. Clin. Investig.* **131**, e143226 (2021). 33463535 10.1172/JCI143226 PMC7810481

[8] Teixeira, A. F., Ten Dijke, P. & Zhu, H.-J. On-target anti-TGF-β therapies are not succeeding in clinical cancer treatments: what are remaining challenges?. *Front. Cell Dev. Biol.* **8**, 605 (2020). 32733895 10.3389/fcell.2020.00605 PMC7360684

[9] Biernacka, A., Dobaczewski, M. & Frangogiannis, N. G. TGF-β signaling in fibrosis. *Growth factors* **29**, 196–202 (2011). 21740331 10.3109/08977194.2011.595714 PMC4408550

[10] Györfi, A. H., Matei, A.-E. & Distler, J. H. Targeting TGF-β signaling for the treatment of fibrosis. *Matrix Biol.* **68**, 8–27 (2018). 29355590 10.1016/j.matbio.2017.12.016

[11] Long, Y., Niu, Y., Liang, K. & Du, Y. Mechanical communication in fibrosis progression. *Trends Cell Biol.* **32**, 70–90 (2022). 34810063 10.1016/j.tcb.2021.10.002

[12] Li, H. et al. Src family kinases and pulmonary fibrosis: a review. *Biomed. Pharmacother.* **127**, 110183 (2020). 32388241 10.1016/j.biopha.2020.110183

[13] Massagué, J. & Sheppard, D. TGF-β signaling in health and disease. *Cell* **186**, 4007–4037 (2023). 37714133 10.1016/j.cell.2023.07.036 PMC10772989

[14] Frangogiannis, N. G. Transforming growth factor-β in myocardial disease. *Nat. Rev. Cardiol.* **19**, 435–455 (2022). 34983937 10.1038/s41569-021-00646-w

[15] Loeys, B. L. et al. Aneurysm syndromes caused by mutations in the TGF-β receptor. *N. Engl. J. Med.* **355**, 788–798 (2006). 16928994 10.1056/NEJMoa055695

[16] Basu, S., Hodgson, G., Katz, M. & Dunn, A. R. Evaluation of role of G-CSF in the production, survival, and release of neutrophils from bone marrow into circulation. *Blood* **100**, 854–861 (2002). 12130495 10.1182/blood.v100.3.854

[17] Hamilton, J. A. Colony-stimulating factors in inflammation and autoimmunity. *Nat. Rev. Immunol.* **8**, 533–544 (2008). 18551128 10.1038/nri2356

[18] Guo, B. et al. CSF3 aggravates acute exacerbation of pulmonary fibrosis by disrupting alveolar epithelial barrier integrity. *Int. Immunopharmacol.* **135**, 112322 (2024). 38788452 10.1016/j.intimp.2024.112322

[19] Adachi, K. et al. Granulocyte colony-stimulating factor exacerbates the acute lung injury and pulmonary fibrosis induced by intratracheal administration of bleomycin in rats. *Exp. Toxicol. Pathol.* **53**, 501–510 (2002). 11926293 10.1078/0940-2993-00218

[20] Henderson, N. C., Rieder, F. & Wynn, T. A. Fibrosis: from mechanisms to medicines. *Nature* **587**, 555–566 (2020). 33239795 10.1038/s41586-020-2938-9 PMC8034822

[21] Margraf, A., Lowell, C. A. & Zarbock, A. Neutrophils in acute inflammation: current concepts and translational implications. *Blood* **139**, 2130–2144 (2022). 34624098 10.1182/blood.2021012295 PMC9728535

[22] Ramaiah, S. K. & Jaeschke, H. Role of neutrophils in the pathogenesis of acute inflammatory liver injury. *Toxicol. Pathol.* **35**, 757–766 (2007). 17943649 10.1080/01926230701584163

[23] Mehta, H. M., Malandra, M. & Corey, S. J. G-CSF and GM-CSF in neutropenia. *J. Immunol.* **195**, 1341–1349 (2015). 26254266 10.4049/jimmunol.1500861 PMC4741374

[24] Lee, J.-U. et al. Granulocyte colony-stimulating factor in bronchoalveolar lavage fluid is a potential biomarker for prognostic prediction of idiopathic pulmonary fibrosis. *Korean J. Intern. Med.* **37**, 979 (2022). 35730133 10.3904/kjim.2021.442 PMC9449205

[25] Zhu, W., Liu, C., Tan, C. & Zhang, J. Predictive biomarkers of disease progression in idiopathic pulmonary fibrosis. *Heliyon* **10**, e23543 (2023). 38173501 10.1016/j.heliyon.2023.e23543 PMC10761784

[26] Tsantikos, E. et al. Granulocyte-CSF links destructive inflammation and comorbidities in obstructive lung disease. *J. Clin. Investig.* **128**, 2406–2418 (2018). 29708507 10.1172/JCI98224 PMC5983324

[27] Richeldi, L. et al. Efficacy and safety of nintedanib in idiopathic pulmonary fibrosis. *N. Engl. J. Med.* **370**, 2071–2082 (2014). 24836310 10.1056/NEJMoa1402584

[28] King, T. E. Jr et al. A phase 3 trial of pirfenidone in patients with idiopathic pulmonary fibrosis. *N. Engl. J. Med.* **370**, 2083–2092 (2014). 24836312 10.1056/NEJMoa1402582

[29] Zheng, Q. et al. Mortality and survival in idiopathic pulmonary fibrosis: a systematic review and meta-analysis. *ERJ Open Res.* **8**, 00591-2021 (2022). 35295232 10.1183/23120541.00591-2021 PMC8918939

[30] Maher, T. M. et al. Global incidence and prevalence of idiopathic pulmonary fibrosis. *Respir. Res.* **22**, 197 (2021). 34233665 10.1186/s12931-021-01791-z PMC8261998

[31] Rout-Pitt, N., Farrow, N., Parsons, D. & Donnelley, M. Epithelial mesenchymal transition (EMT): a universal process in lung diseases with implications for cystic fibrosis pathophysiology. *Respir. Res.* **19**, 1–10 (2018). 30021582 10.1186/s12931-018-0834-8 PMC6052671

[32] Bobis-Wozowicz, S. & Paw, M. Transcriptional orchestration of EMT: unraveling novel molecular targets in pulmonary fibrosis. *Mol. Ther.* **32**, 3765–3767 (2024). 39489906 10.1016/j.ymthe.2024.10.012 PMC11573601

[33] Sisto, M. & Lisi, S. Epigenetic regulation of EMP/EMT-dependent fibrosis. *Int J. Mol. Sci.* **25**, 2775 (2024). 38474021 10.3390/ijms25052775 PMC10931844

[34] Welte, K. et al. Recombinant human granulocyte colony-stimulating factor. Effects on hematopoiesis in normal and cyclophosphamide-treated primates. *J. Exp. Med.* **165**, 941–948 (1987). 3494094 10.1084/jem.165.4.941 PMC2188574

[35] Tsioumpekou, M., Krijgsman, D., Leusen, J. H. & Olofsen, P. A. The role of cytokines in neutrophil development, tissue homing, function and plasticity in health and disease. *Cells* **12**, 1981 (2023). 37566060 10.3390/cells12151981 PMC10417597

[36] Mack, M. Inflammation and fibrosis. *Matrix Biol.* **68-69**, 106–121 (2018). 29196207 10.1016/j.matbio.2017.11.010

[37] Correa-Gallegos, D., Jiang, D. & Rinkevich, Y. Fibroblasts as confederates of the immune system. *Immunol. Rev.* **302**, 147–162 (2021). 34036608 10.1111/imr.12972

[38] Mehta, H. M. & Corey, S. J. G.-C. S. F. the guardian of granulopoiesis. *Semin. Immunol.* **54**, 101515 (2021). 34772606 10.1016/j.smim.2021.101515

[39] Zhu, Y. et al. Interplay between extracellular matrix and neutrophils in diseases. *J. Immunol. Res.* **2021**, 8243378 (2021). 34327245 10.1155/2021/8243378 PMC8302397

[40] Strøbech, J. E., Giuriatti, P. & Erler, J. T. Neutrophil granulocytes influence on extracellular matrix in cancer progression. *Am. J. Physiol.* **323**, C486–C493 (2022). 10.1152/ajpcell.00122.2022 35759433

[41] Mousset, A. et al. Neutrophil extracellular traps formed during chemotherapy confer treatment resistance via TGF-β activation. *Cancer Cell* **41**, 757–775.e710 (2023). 37037615 10.1016/j.ccell.2023.03.008 PMC10228050

[42] Martins, A., Han, J. & Kim, S. O. The multifaceted effects of granulocyte colony-stimulating factor in immunomodulation and potential roles in intestinal immune homeostasis. *IUBMB life* **62**, 611–617 (2010). 20681025 10.1002/iub.361 PMC2916186

[43] Roberts, A. W. G-CSF: a key regulator of neutrophil production, but that’s not all!. *Growth Factors* **23**, 33–41 (2005). 16019425 10.1080/08977190500055836

[44] Jiang, H.-M. et al. Role for granulocyte colony stimulating factor in angiotensin II-induced neutrophil recruitment and cardiac fibrosis in mice. *Am. J. Hypertens.* **26**, 1224–1233 (2013). 23761490 10.1093/ajh/hpt095

[45] Sugimoto, C. et al. Granulocyte colony-stimulating factor (G-CSF)-mediated signaling regulates type IV collagenase activity in head and neck cancer cells. *Int. J. Cancer* **93**, 42–46 (2001). 11391619 10.1002/ijc.1297

[46] Fernandez, I. E. & Eickelberg, O. The impact of TGF-beta on lung fibrosis: from targeting to biomarkers. *Proc. Am. Thorac. Soc.* **9**, 111–116 (2012). 22802283 10.1513/pats.201203-023AW

[47] Frangogiannis, N. Transforming growth factor-beta in tissue fibrosis. *J. Exp. Med.* **217**, e20190103 (2020). 32997468 10.1084/jem.20190103 PMC7062524

[48] Scotton, C. J. & Chambers, R. C. Molecular targets in pulmonary fibrosis: the myofibroblast in focus. *Chest* **132**, 1311–1321 (2007). 17934117 10.1378/chest.06-2568

[49] Fernandez, I. E. & Eickelberg, O. The impact of TGF-β on lung fibrosis: from targeting to biomarkers. *Proc. Am. Thorac. Soc.* **9**, 111–116 (2012). 22802283 10.1513/pats.201203-023AW

[50] Deng, Z. et al. TGF-β signaling in health, disease, and therapeutics. *Signal Transduct. Target Ther.* **9**, 61 (2024). 38514615 10.1038/s41392-024-01764-w PMC10958066

[51] Akhurst, R. J. & Hata, A. Targeting the TGFβ signalling pathway in disease. *Nat. Rev. Drug Discov.* **11**, 790–811 (2012). 23000686 10.1038/nrd3810 PMC3520610

[52] Shi, R. et al. Collagen prolyl 4-hydroxylases modify tumor progression. *Acta Biochim. Biophys. Sin. (Shanghai)* **53**, 805–814 (2021). 34009234 10.1093/abbs/gmab065

[53] Martinez, F. J. et al. Idiopathic pulmonary fibrosis. *Nat. Rev. Dis. Prim.* **3**, 17074 (2017). 29052582 10.1038/nrdp.2017.74

[54] Herrera, J. A. et al. The UIP/IPF fibroblastic focus is a collagen biosynthesis factory embedded in a distinct extracellular matrix. *JCI Insight* **7**, e156115 (2022). 35852874 10.1172/jci.insight.156115 PMC9462507

[55] Seluanov, A., Vaidya, A. & Gorbunova, V. Establishing primary adult fibroblast cultures from rodents. *J. Vis. Exp.* **5**, 2033 (2010). 10.3791/2033 PMC3185624 20972406

[56] Varghese, F., Bukhari, A. B., Malhotra, R. & De, A. IHC Profiler: an open source plugin for the quantitative evaluation and automated scoring of immunohistochemistry images of human tissue samples. *PLoS ONE* **9**, e96801 (2014). 24802416 10.1371/journal.pone.0096801 PMC4011881

