# Supplementary material for: Colony-stimulating factor 3 as a key mediator in the progression of idiopathic pulmonary fibrosis: a novel therapeutic target
Source: Signal Transduct Target Ther. 2025 Oct 2;10:322. doi: 10.1038/s41392-025-02421-6 (PMC12489044; doi:10.1038/s41392-025-02421-6)
Supplement: Supplementary file 1 — Quality check Supplementary Figure 1-12 - Clean version [file 41392_2025_2421_MOESM1_ESM.docx]

Supplementary Materials for

Colony-Stimulating Factor 3 as a Key Mediator in the Progression of Idiopathic Pulmonary Fibrosis: A Novel Therapeutic Target

Seungmo Kim^1*^, Yongjoong Kim^1*^, Tae-Sung Kim^1^, Jae-Hyeok Kang^2^, In-Yeong Yun^2^, Eung-Suk Lee^1^, Eun Ji Lee^1^, Rae-kwon Kim^3^, Joo Mi Yi^4,5^, Hye Sook Choi^6^, Jin Woo Song^7^, Young Woo Jin^1#^, Min-Jung Kim^1#^ & Su-Jae Lee^1#^.

Correspondence to: ywjin@fnctbiotech.com (Y.W.J.); mjkim@fnctbiotech.com (M.J.K.); sjlee@fnctbiotech.com (S.J.L.)

**This PDF file includes:**

Materials and Methods

Supplementary Figure S1 to S12

Table S1

**Materials and Methods**

**Cell culture and treatments**

The cell lines used in this study were obtained or purchased from Korean Cell Line Bank (KCLB) and American Type Culture Collection (ATCC). IPF patient-derived fibroblast cells (IPDF), adult human normal primary lung fibroblast cells (AHLF) and human fetal lung fibroblast cell line (MRC5) were cultured in DMEM (Life Technologies, 10938025), respectively. All culture media were supplemented with 10 % Fetal Bovine Serum (Gibco™ FBS, Life Technologies, 16000044) and 1 % Antibiotic/Antimycotic solution (Gibco™ 15240062). The cells were incubated at 37 °C in a 5 % CO2 humidified atmosphere. In order to ascertain the inhibitory effect of the antibody on the myofibroblast transition of fibroblasts induced by recombinant human TGF-β1 (rhTGF-β1), cells were seeded in 60 mm plates at a density of approximately 3 x 10⁵ (MRC5) or 2.5 x 10⁵ (IPDF, AHLF) cells. Following a 16-hour incubation period, the medium was replaced with FBS-free medium and, two hours later, the cells were treated with the indicated concentrations of antibody. After one hour, the cells were treated with recombinant TGF-β1 protein (2 ng/ml) and the cells were harvested for analysis 24 hours later.

**Western blot analysis**

Cell lysates were prepared by extracting proteins on ice with TNN lysis buffer (40mM Tris-HCl, pH 8.0, 120 mM NaCl, 0.1% Nonidet-P40) supplemented with protease inhibitor cocktail (GenDEPOT). Proteins were separated by SDS-PAGE and transferred to a nitrocellulose membrane (Amersham, #10600002). To detect protein bands, membranes were incubated with enhanced chemiluminescence (ECL) and visualized with an Amersham ImageQuant 800 (GE Healthcare). Western blot quantification was performed by quantifying each sample via densitometric analysis with ImageJ, then normalizing to β-actin to allow relative comparison of different samples.

**Quantitative RT-PCR**

Total RNA was isolated from cells or lung tissue manually using TRIzol reagent (Invitrogen, #15596026) according to the manufacturer's protocol. Briefly, cDNA was synthesized using 1 μg of total RNA using the SuperScript™ III First-Strand Synthesis System (Invitrogen). qRT-PCR reactions were performed on CFX Duet (BioRad) using the SensiFAST™ SYBR® No-ROX Kit (Bioline, #BIO-98002), and results were expressed as fold change calculated with the ΔΔCt method relative to the control sample. Target gene expression in each sample was normalized to the mRNA level of 18S rRNA, an internal control. Primer sequences used in this study are listed in Supplementary Table 1.

**Immunocytochemistry**

Cells cultured on coverslips were fixed with 4% paraformaldehyde (PFA) at 20min 4℃. Following fixation, permeabilizing and blocking were performed with PBS containing 0.2% NP-40 and 10% FBS. After blocking, the cells were incubated with a primary antibody with a blocking buffer at 4℃ overnight. After washing the primary antibody, the cells were detected with anti-mouse or anti-rabbit Alex Fluor 488 conjugated secondary antibody. Cell nuclei were counterstained using 4’,6-diamidino-2-phenylindole (DAPI) (Sigma Aldrich). The immunostained cells were then imaged using an IX71 microscope (Olympus).

**In situ proximity ligation assay (PLA)**

Cultured cells were fixed with 4% paraformaldehyde (PFA) at 20min 4℃. Fixed cells were permeabilized with 0.1% Triton X-100 and 10% FBS in PBS. After blocking, cells were incubated with primary antibodies (1:500) at 4℃ overnight. In situ PLA was performed per the manufacturer’s protocol, using a Duolink detection kit with a pair of nucleotide-labeled 2nd antibodies. After ligation and amplification of PLA probes, signals were visualized under a confocal microscopy (Nikon C2 confocal microscope, Nikon) and quantified using Image J software.

**Chromatin immunoprecipitation (ChIP) assays**

Prior to the ChIP assay, cells were cross-linked with final concentration of 1% formaldehyde. The ChIP assay was performed using the EZ-ChIPTM kit (Millipore, # 17-371) according to the manufacturer’s instructions. For immunoprecipitation (IP), anti-phospho-STAT3 (cell signaling, 9145S, 1:50) and anti-phospho-SMAD2/3(cell signaling, 8828S, 1:50), and anti-mouse IgG as negative controls. The transcription factor binding target binding of STAT3, and SMAD2/3 promoter region was predicted by JASPAR (https://jaspar.elixir.no) and UCSC Genome Browser (https://genome.ucsc.edu/index.html). The primer sequences used in the ChIP assay are presented in Supplementary Table 1. For the normalization of ChIP-qPCR data, two common methods are used percentage of input and the fold enrichment. The percentage of Input was calculated based on the equation 100 x 2Ct (input)-Ct (Ip). The fold enrichment normalized to the Ct of IgG.

**Chemical reagents and antibodies**

Bleomycin Sulfate were purchased from AdooQ Bioscience (#A10152) and Sigma-Aldrich (#B1141000). Recombinant TGF-β1 systems (human; #240-B-CS, mouse; #7666-MB) and CSF3 (human; #214-CS, mouse; #414-CS were purchased from R&D. STAT3 Inhibitor III (WP1066, #57-309-7) and PI-3K inhibitor (LY294002, #44-020) were purchased from Calbiochem. Antibodies to Alpha-smooth muscle actin (α-SMA, #ab5694, #ab7817), Collagen I (#ab34710), Fibronectin (#ab2413), Versican (#ab177480), G-CSF (#ab9691, #ab181053), TGF-β1 (#ab92486), CD31 (#ab281583), MPO (#ab208670), Surfactant protein A (#ab115791), pro-Surfactant protein C (#ab40879) antibody were purchased from Abcam. Snail (#3879), Slug (#9585), Vimentin (#5741), p-STAT3 (Y705; #4113, #9145), p-Smad3 (Ser423/425; #9520), p-AKT (S473; #4060), and HRP-conjugated secondary antibodies (#7076, #7074) were purchased from Cell signaling Technology. β-actin (#sc-47778), and CD45 (#sc-1178) antibody was purchased from SantaCruz Biotechnology. E-cadherin (#610182) and N-cadherin (#610920) antibodies were purchased from BD Transduction Laboratories. CD86 (#PA5-114995) antibody was purchased from Invitrogen. Biotinylated anti-mouse/rabbit/goat IgG antibody was purchased from Vector laboratories (#BA-1300-2.2). Has3 (#PA5-100552) and Vimentin (#3634-100) antibodies were purchased from Invitrogen and BioVision Inc, respectively.

**Enzyme-linked immunosorbent assay (ELISA)**

The levels of CSF3 and TGF-β1 in human or mouse blood serum, mouse lung tissue, cell lysates or culture supernatants were detected by enzyme-linked immunosorbent assay (ELISA) kits following the manufacturer’s instructions. Quantikine TGF-β1 (#DB100C), Human G-CSF (#DCS50), and Mouse G-CSF (#MCS00) were purchased from R&D systems.

**Matrigel-invasion assay**

For Matrigel-invasion assay was seeded on Transwell chambers (8μm pore size; Corning, 3422). The Transwell chamber membranes were pre-coated with growth factor-reduced Matrigel (BD Biosciences, CLS356231). The lower chamber was filled with 800 ul of growth medium. Non-invading cells were cleared using a cotton swab. Invading cells were fixed with 4% paraformaldehyde (Biosesang, PC2031-100-00), stained with crystal violet (Sigma-Aldrich, C6158), and photographed using an IX71 microscope. The number of cells in three microscopic fields from each Transwell was counted.

**Supplementary Figure and Legends**

**
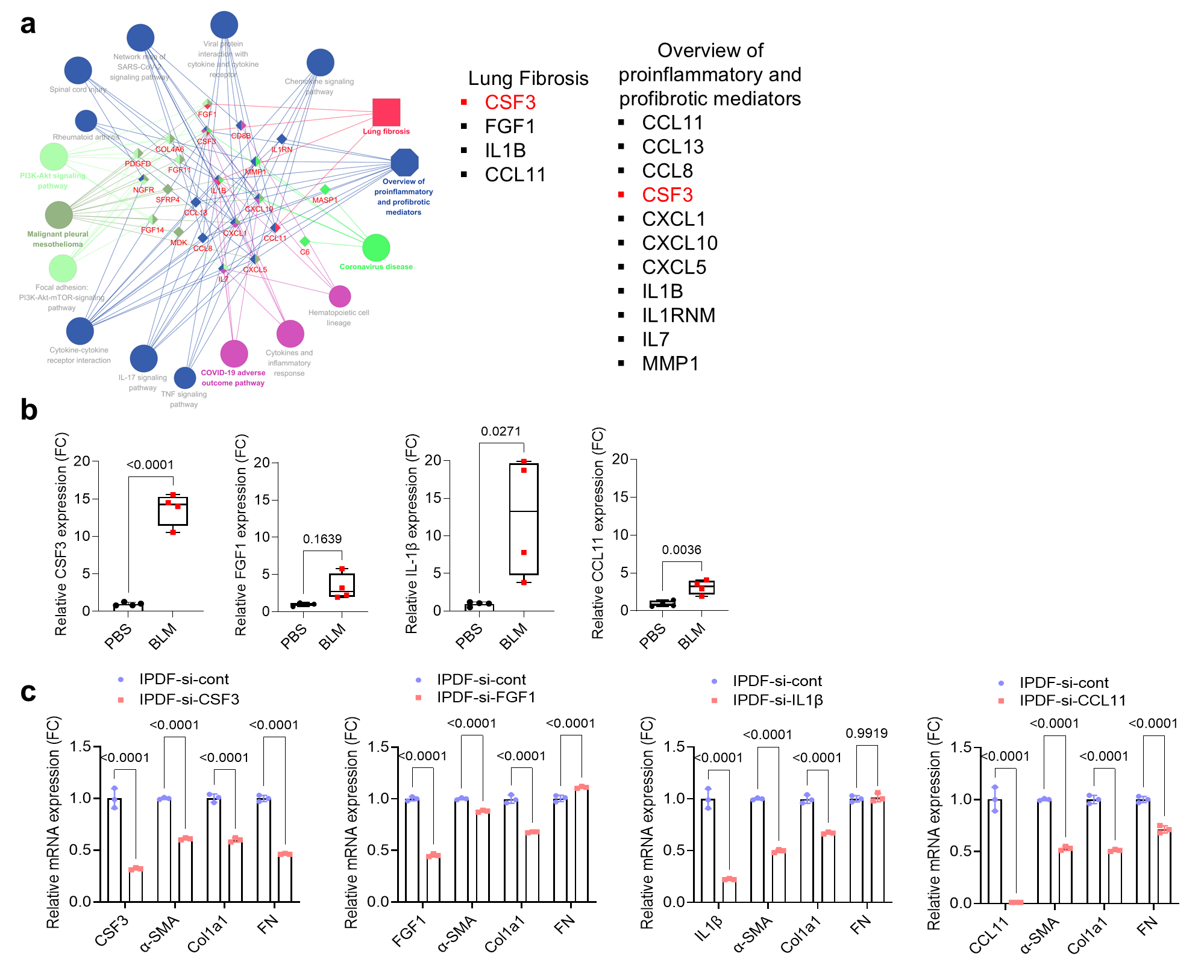
**

**Supplementary Figure. 1.**

**a,** A network analysis of lung fibrosis-related genes was performed using Cytoscape. Lung fibrosis-related genes (CSF3, FGF1, IL1β, CCL11) were identified from co-elevated genes in GSE134692 and GSE71351 datasets, where the co-elevated genes exhibited a 1.5-fold increase in IPF patients compared to normal subjects. **b****,** qRT-PCR analysis of fibrosis-related gene expression in mouse lung tissue from intratracheal injection (I.T) bleomycin-induced pulmonary fibrosis model (n = 4 mice per group). Statistical significance was determined using t-test. **c,** qRT-PCR analysis was conducted to assess the expression of fibrosis markers in IPDF that were transfected with target siRNAs (si-CSF3, si-FGF1, si-IL1β, si-CCL11). Statistical significance was determined using t-test.

**
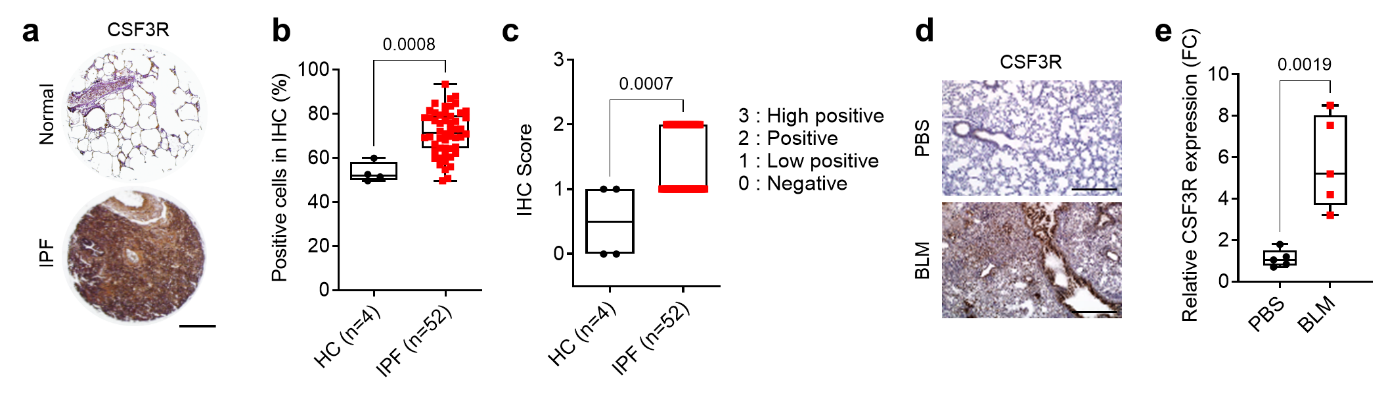
**

**Supplementary Figure. 2.**

**a,** Immunohistochemical (IHC) staining of CSF3R in a human pulmonary fibrosis tissue array (LC561a). **b-c,** CSF3R IHC scoring of human pulmonary fibrosis tissue array using two distinct IHC profiler-based analytical approaches (healthy control (HC) = 4, IPF = 52 case). Scale bar = 500 μm. **d-e,** IHC analysis of CSF3R (**d**) and qRT-PCR analysis of CSF3R expression (**e**) in mouse lung tissue from bleomycin-induced pulmonary fibrosis model. (n = 5 mic per group). Statistical significance was determined using t-test.


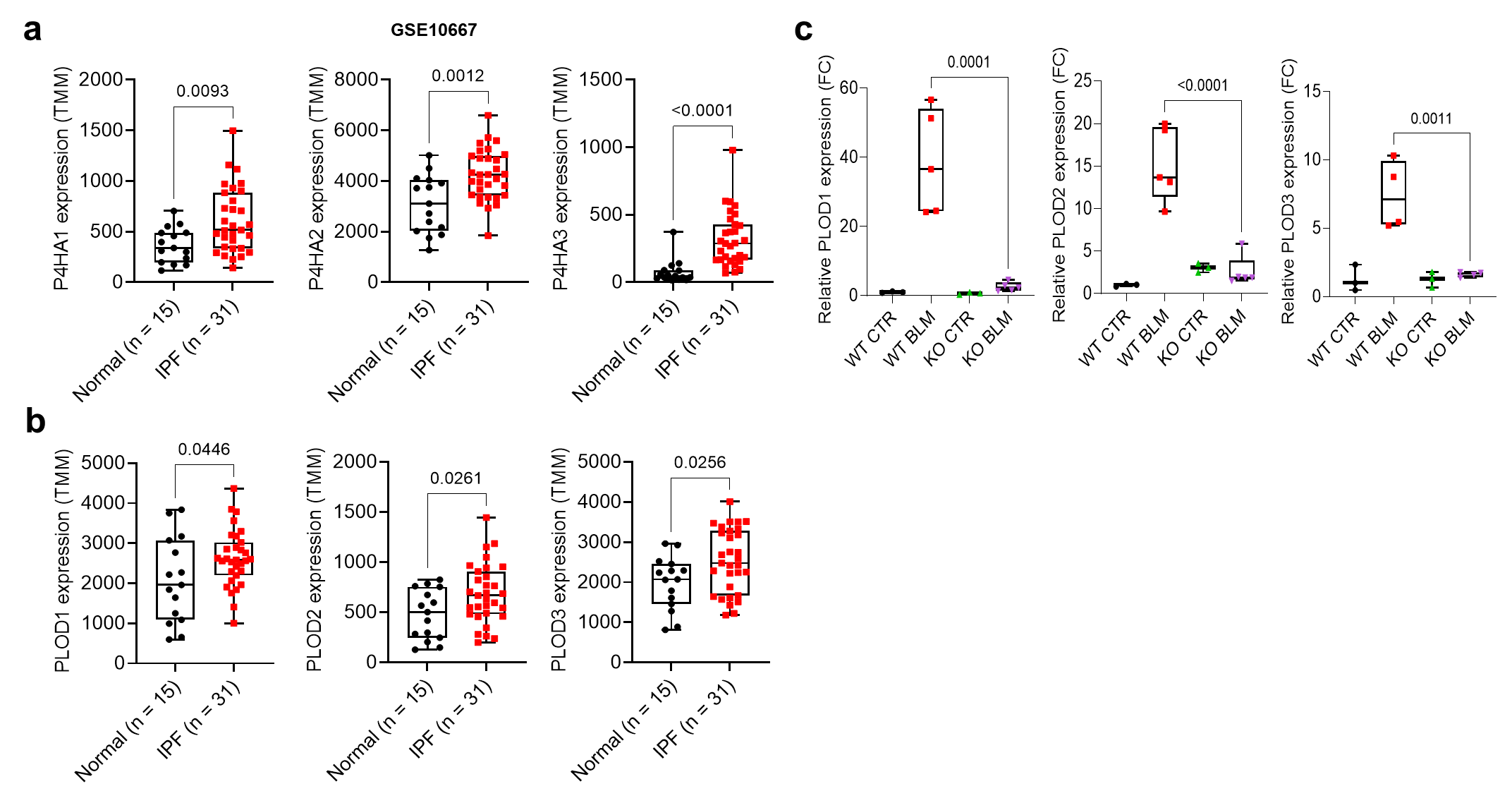


**Supplementary Figure. 3.**

**a-b,** The expression levels of the prolyl hydroxylases (P4HA1, P4HA2, P4HA3) and lysyl hydroxylases (PLOD1, PLOD2, PLOD3) in both normal and IPF patient groups (GSE10667). **c,** qRT-PCR analysis of lysyl hydroxylases expression in lung tissue from CSF3 wild-type (CSF3^+/+^) and CSF3 knockout (CSF3^-/-^) mice after BLM or PBS treatment. Statistical significance was determined using ANOVA with multiple comparison or t-test.


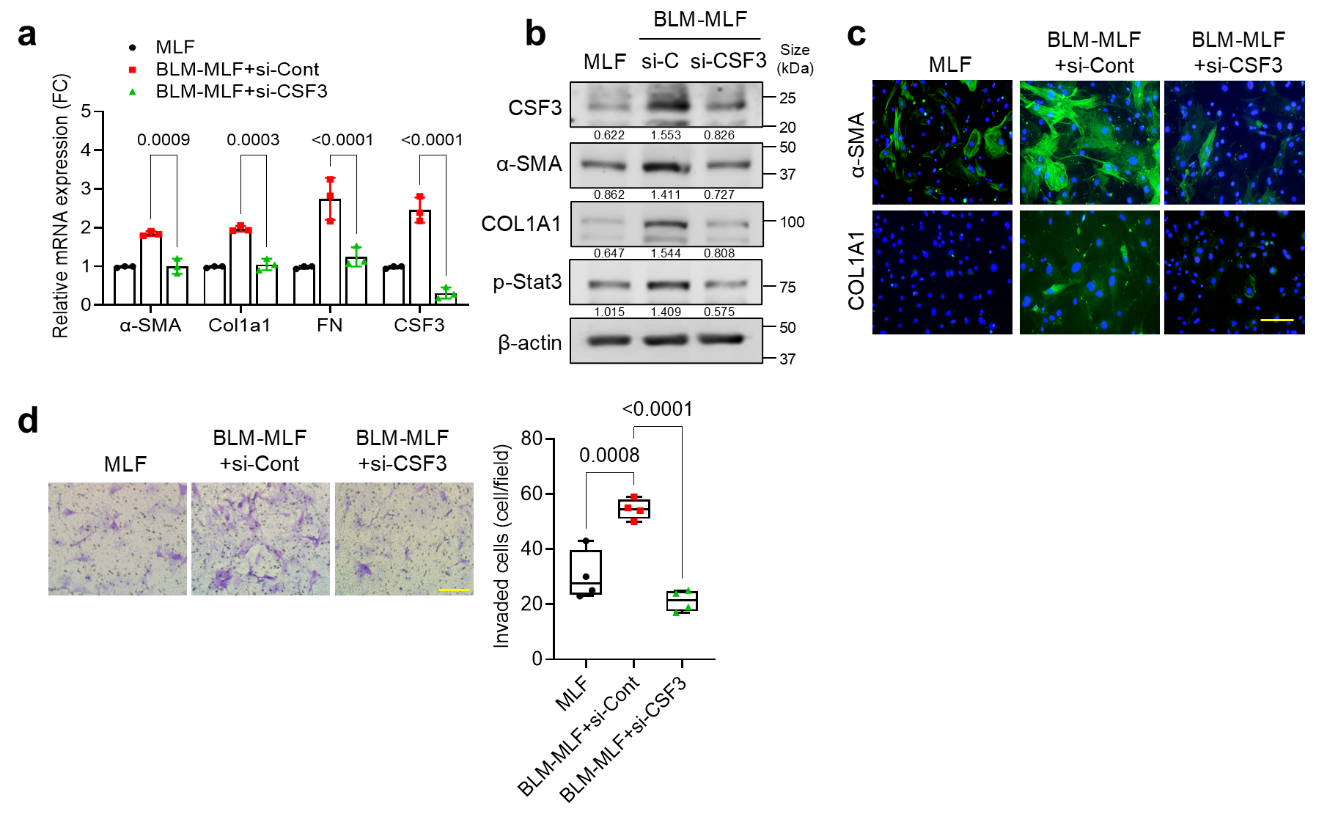


**Supplementary Figure. 4.**

**a-c,** qRT-PCR analysis of fibrosis markers (α-SMA, COL1A1, and fibronectin (FN)) and CSF3 (**a**), and Western blot analysis of CSF3, α-SMA, COL1A1, and phospho-STAT3 expression (**b**), and representative immunofluorescence images of α-SMA and COL1A1 (**c**) in MLF and BLM-induced IPF mouse lung fibroblasts (BLM-MLF) transfected with si-control or si-CSF3. β-Actin was used as a loading control. Scale bars: 200 μm. **d,** The spontaneous Matrigel-invading capacity of MLF and BLM-MLF transfected with si-control or si-CSF3. Scale bars: 200 μm. Statistical significance was determined using ANOVA with multiple comparison.


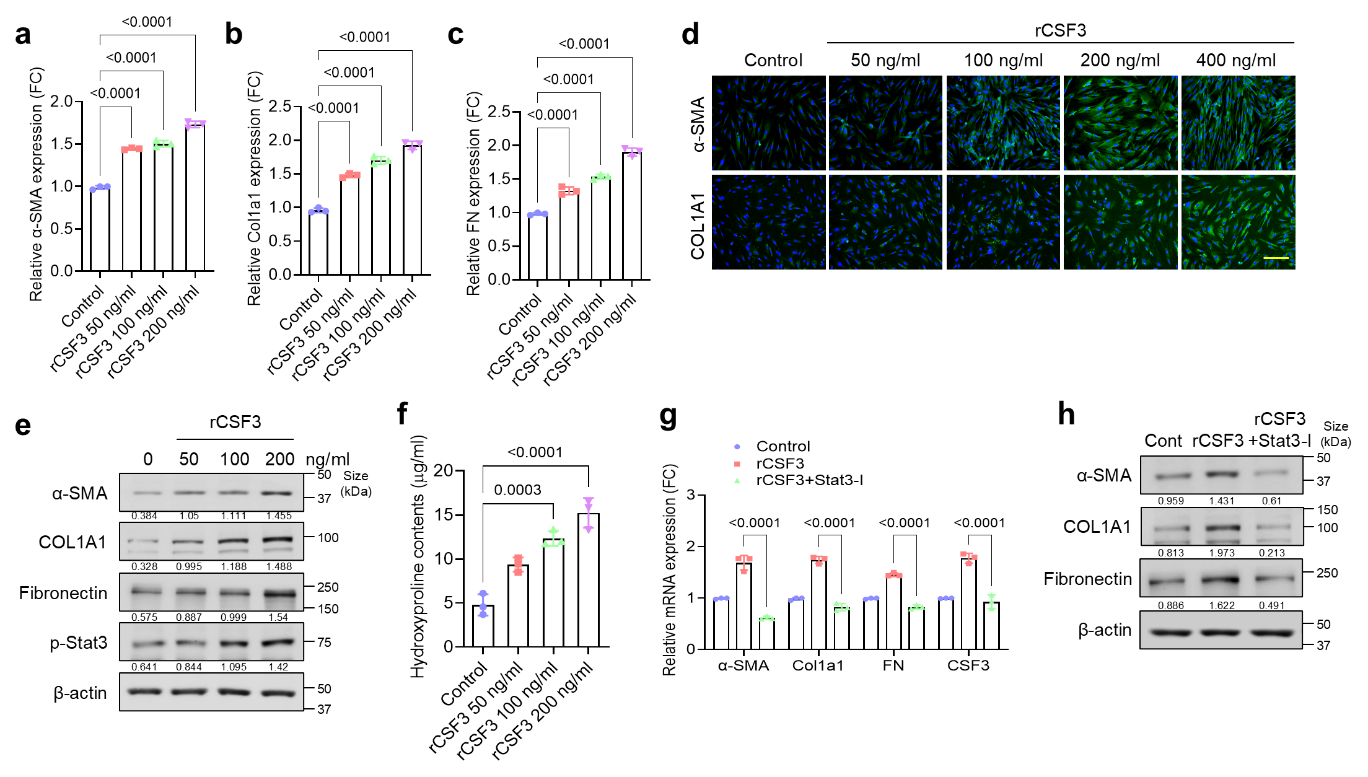


**Supplementary Figure. 5.**

**a-c,** qRT-PCR analysis of the expression of fibrosis markers (α-SMA, COL1A1, and fibronectin (FN)) in HLF treated with increasing doses of CSF3 (50-200 ng/ml, 2 h). **d,** Representative immunofluorescence images of α-SMA and COL1A1 immunostaining of HLF treated with increasing doses of CSF3 (50-200 ng/ml, 24 h). Scale bars: 200 μm. **e,** Western blot analysis of fibrosis markers and phospho-STAT3 in HLF cells treated with increasing doses of CSF3 (50-200 ng/ml, 24 h). β-actin used as a loading control. **f,** Hydroxyproline content in HLF cells treated with increasing doses of CSF3 (50-200 ng/ml, 24 h). **g,** qRT-PCR analysis of fibrosis markers and CSF3 expression in HLF cells treated with recombinant human CSF3 and a STAT3 inhibitor (WP1066), as indicated. **h,** Western blot analysis of fibrosis markers in HLF cells treated with recombinant CSF3 and WP1066, as indicated. Statistical significance was determined using ANOVA with multiple comparison.


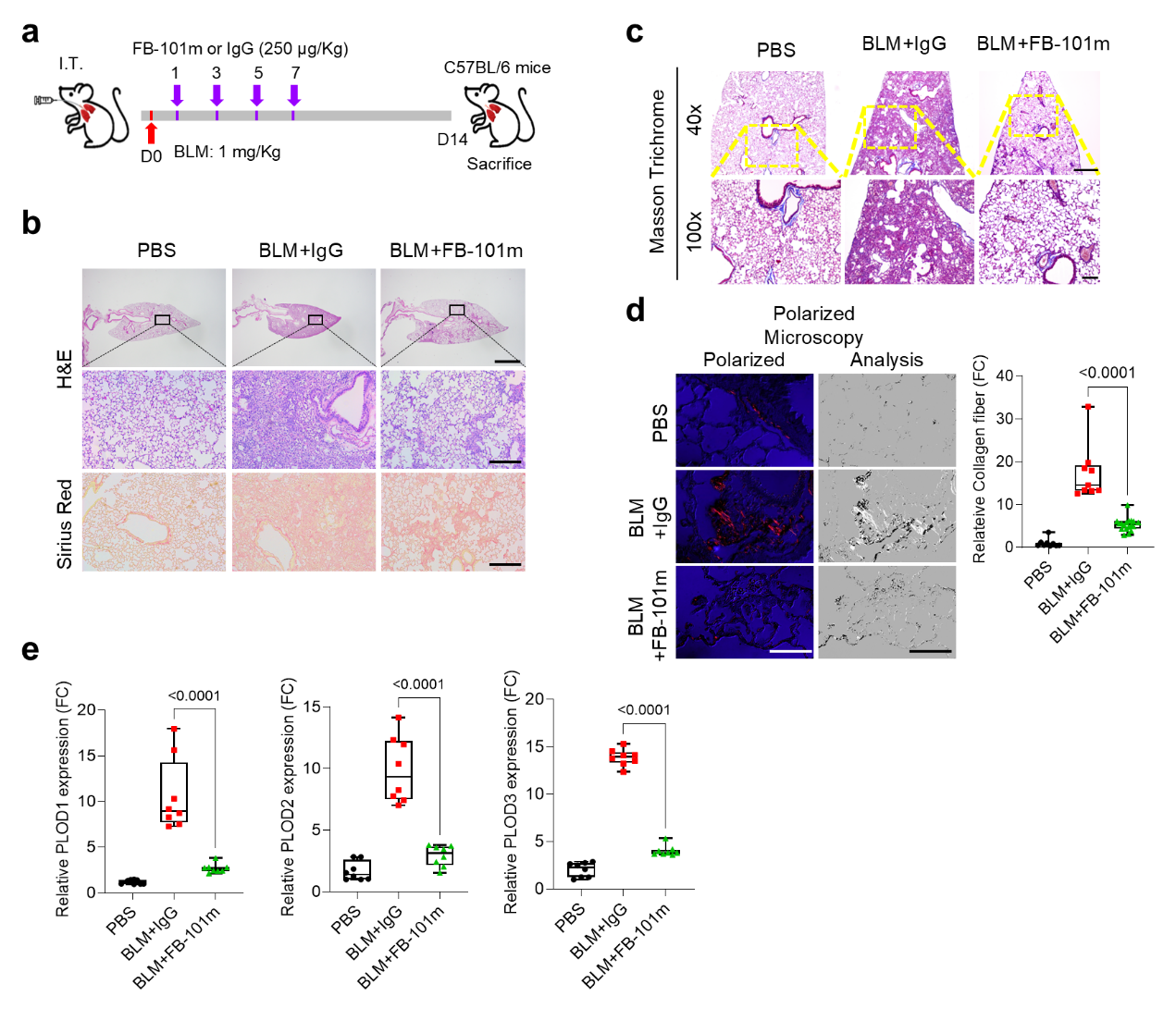


**Supplementary Figure. 6.**

**a,** Schematic illustration of the BLM I.T injection animal model, the generation of lung fibrosis, and the overall procedure for inhibiting fibrosis in C57BL/6 mice. FB-101m and IgG were administered intraperitoneally. **b,** Representative images of H&E and Picrosirius red staining of lung tissue from each group (n=5, each group). Scale bar: 2μm, 250 μm. **c,** Representative Masson's trichrome staining image of lung tissue from each group (n=5, each group). 40x; Scale bar: 500 μm, 100x; Scale bar 100 μm. **d,** Analysis of Picrosirius red-stained I.T. therapeutic mouse model samples by polarized microscopy. The image captured using polarized light depicts collagen fibers (left). The image generated through analysis using Image J provides a detailed visual representation of the collagen fibers (right). Scale bar: 100 μm. **e,** qRT-PCR analysis of lysyl hydroxylases (PLOD1, PLOD2, PLOD3) expression in each mouse group (n=3, each group). Statistical significance was determined using ANOVA with multiple comparison.


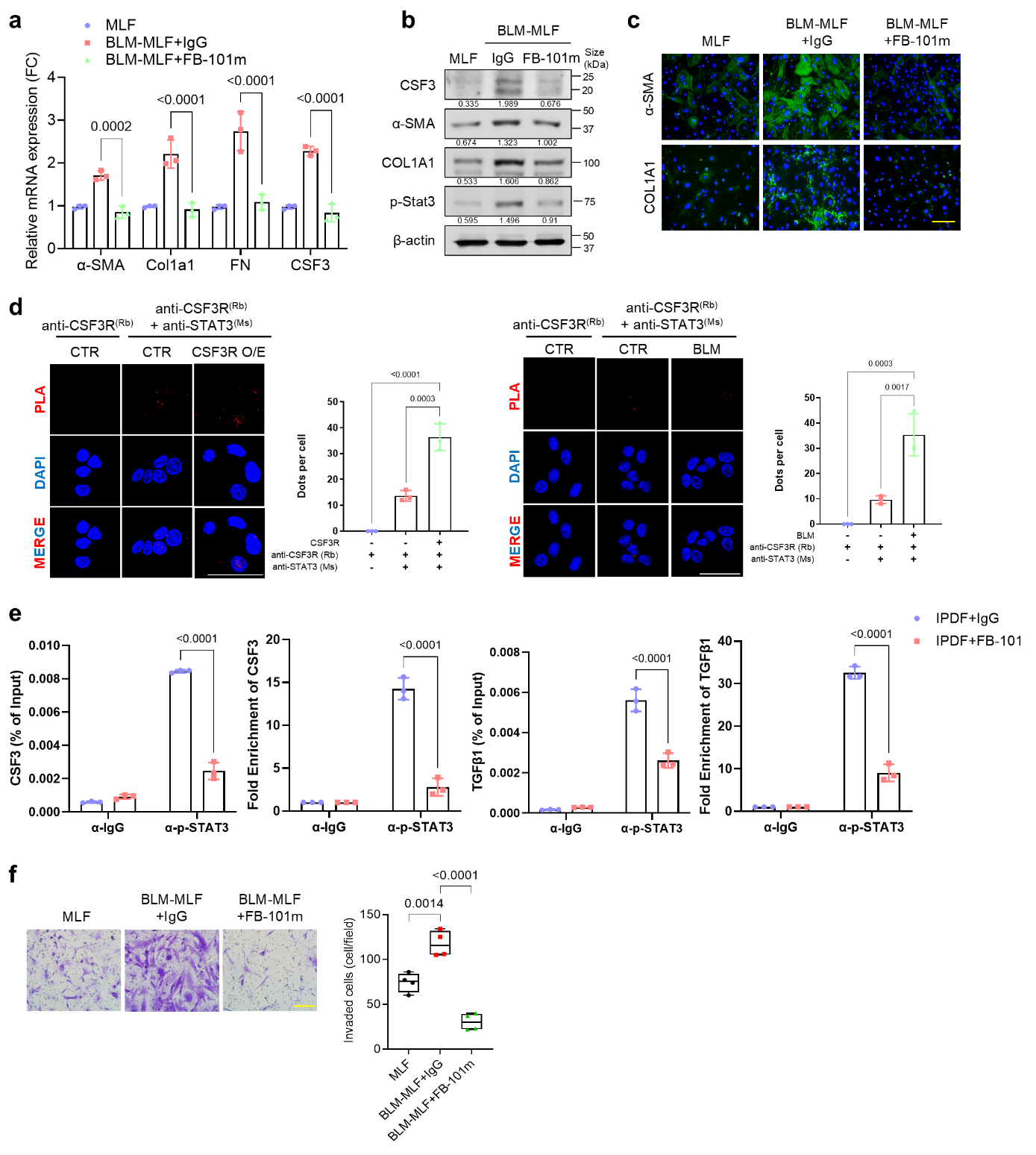


**Supplementary Figure. 7.**

**a-c,** qRT-PCR analysis of fibrosis markers and CSF3 (**a**), and Western blot analysis of CSF3, α-SMA, COL1A1, and phospho-STAT3 levels (**b**), and representative immunofluorescence images of α-SMA and COL1A1 (**c**) in MLF and BLM-MLF treated with IgG or FB-101m. β-Actin was used as a loading control. Scale bars: 200 μm. **d**, Representative images and quantification of in situ PLA showing the interaction between CSF3R and STAT3 in normal human bronchial epithelial cells. Scale bars: 50 μm. **e**. The DNA binding consensus sequence of STAT3 was analyzed by the JASPAR online tool (https://jaspar.elixir.no). Chromatin immunoprecipitation (ChIP)-qPCR analysis showed that phospho-STAT3 can directly bind to the specific site (CTTCTGGGAAA) on the CSF3 and TGF-β1 promoters, which was confirmed to be regulated by FB-101. **f,** The spontaneous Matrigel-invading capacity of MLF and BLM-MLF treated with IgG or FB-101m. Scale bars: 200 μm. Statistical significance was determined using ANOVA with multiple comparison.


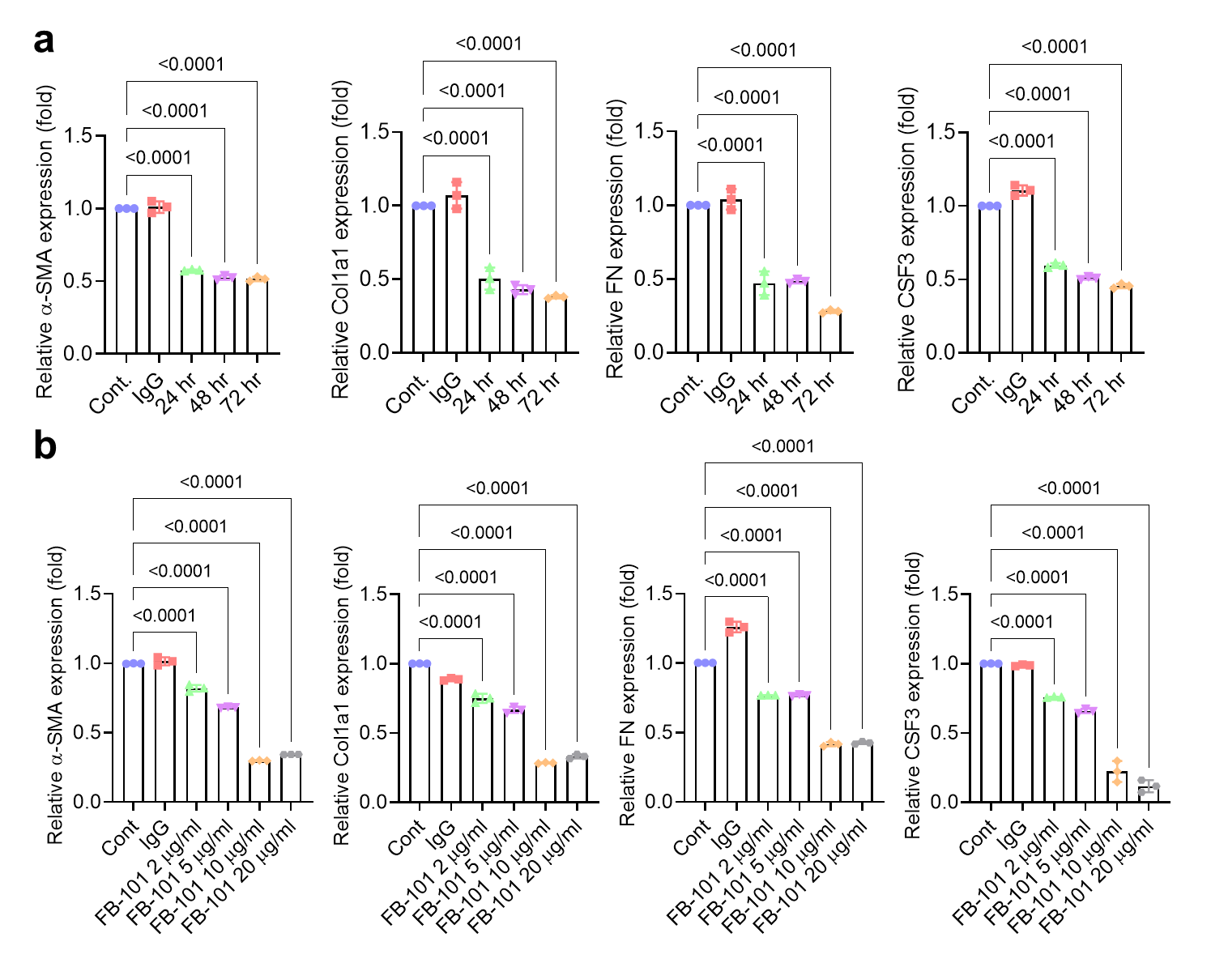


**Supplementary Figure. 8.**

**a-b,** qRT-PCR analysis of fibrosis markers and CSF3 expression according to FB-101 treatment time (**a**) and dose-dependent manner (**b**) in IPDF. Statistical significance was determined using ANOVA with multiple comparison.


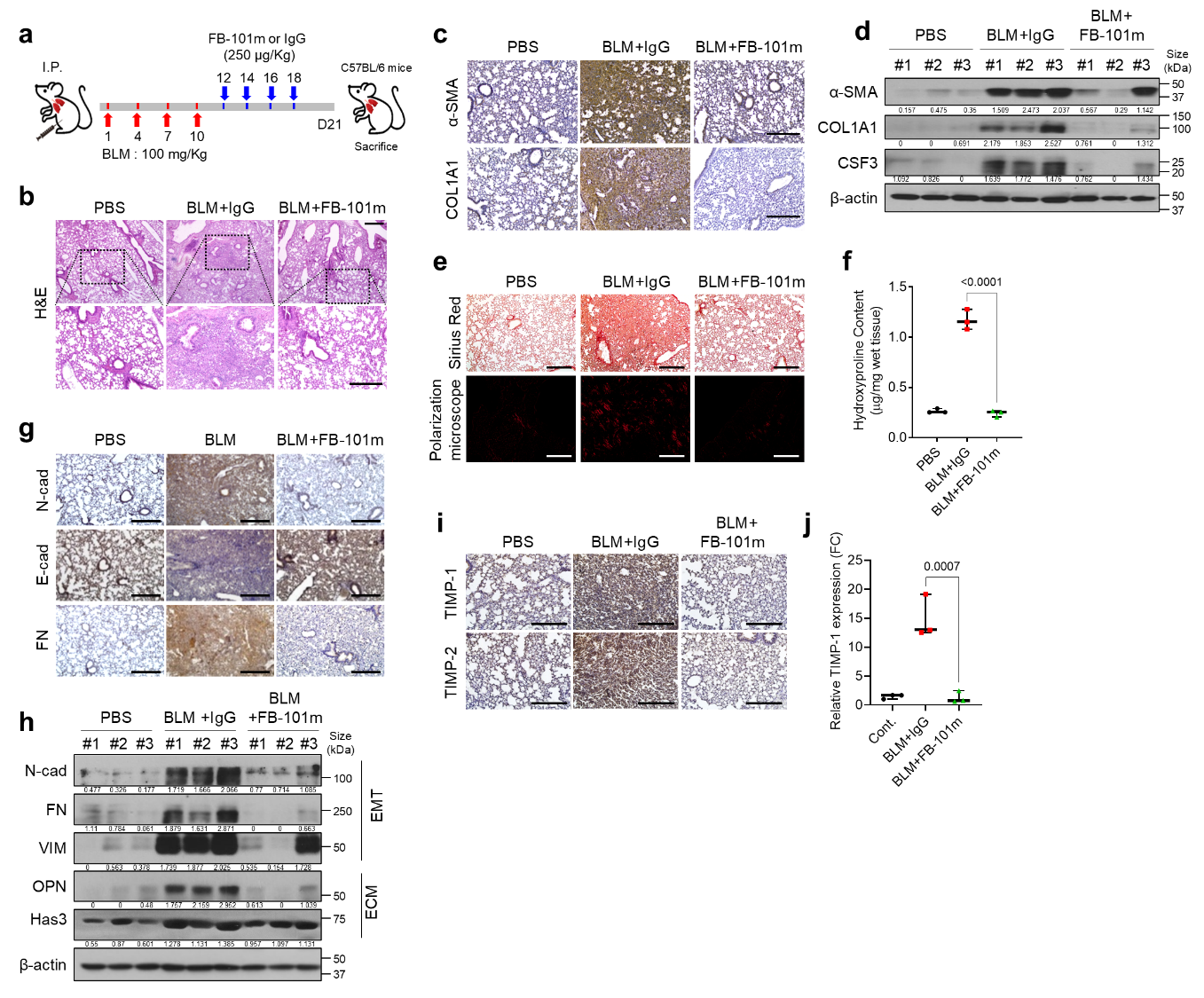


**Supplementary Figure. 9.**

**a,** Schematic illustration of the BLM I.P injection animal model, the generation of lung fibrosis, and the overall fibrosis therapeutic procedure in C57BL/6 mice. FB-101m and IgG were administered via I.P. **b,** Histological examination of mouse lung tissue from each mouse group using the H&E staining. Scale bar: 500 μm, 250 μm. **c,** Representative image of α-SMA and COL1A1 immunostaining in mouse lung tissue from each mouse group. Scale bar: 250 μm. **d,** Western blot analysis of α-SMA, COL1A1, and CSF3 expression in each mouse group (n=3, per group). **e,** Picrosirius red-stained therapeutic mouse model samples analyzed by polarized microscopy. Collagen fibers (left, polarized light) and their subsequent analysis using ImageJ (right, analysis) provide a quantitative assessment of the fibers. Scale bar: 250 μm, 100 μm. **f-h,** Hydroxyproline content assay (**f**), immunohistochemistry for N-cadherin, E- cadherin and FN (**g**), and Western blot analysis of EMT and ECM markers (**h**) in mouse lung tissue from each mouse group (n = 3 per group). Scale bar: 250 μm. **i-j,** Immunohistochemistry for TIMPs (TIMP1/2) (**i**), and qRT-PCR of TIMP1 expression (**j**) in mouse lung tissue from each mouse group (n=3, each group). Scale bar: 250 μm. Statistical significance was determined using ANOVA with multiple comparison.


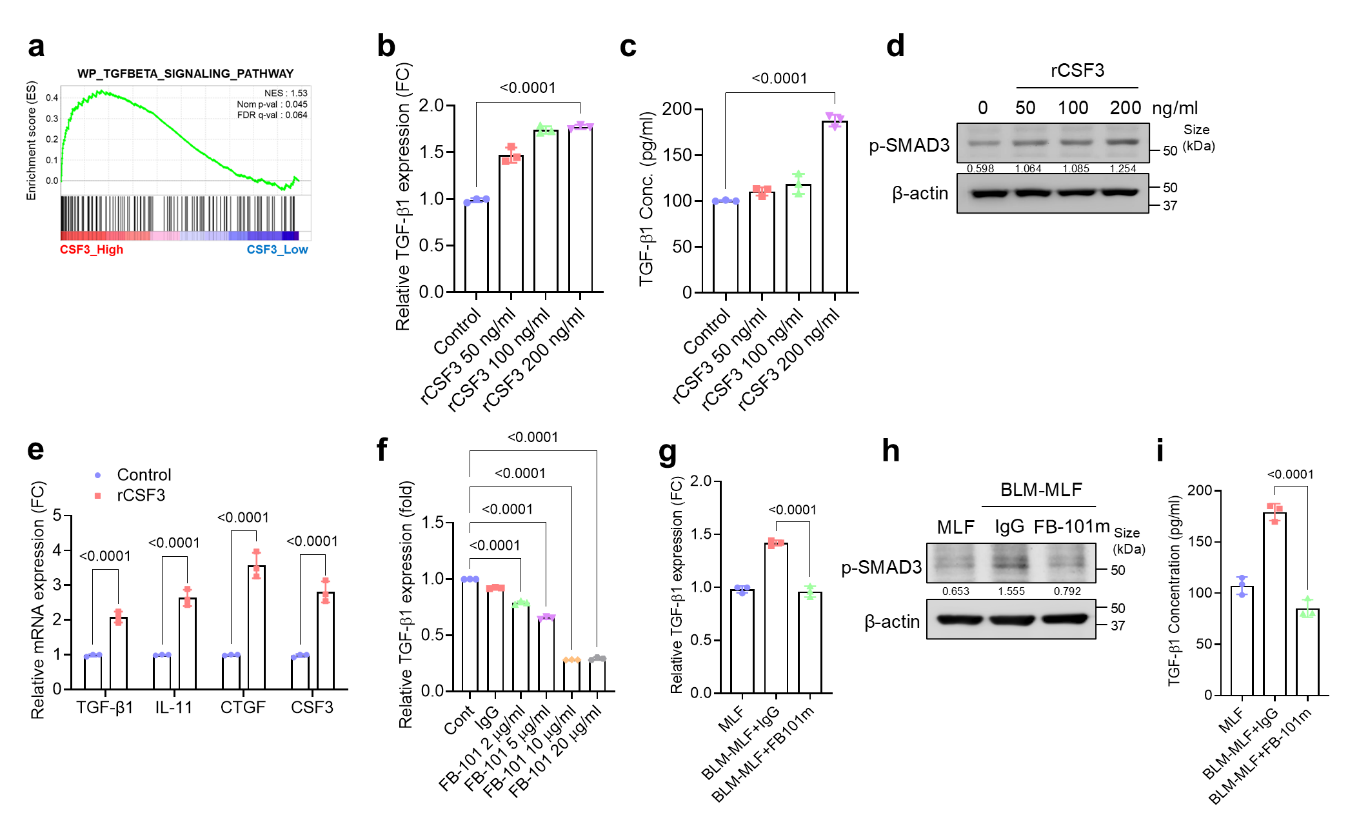


**Supplementary Figure. 10.**

**a,** GSEA for TGF-1β signaling-related significant correlation with high expression of CSF3 in patients (GSE10667). **b-d** qRT-PCR analysis of TGF-β1 expression (**b**), ELISA quantification of TGF-β1 levels (**c**), and Western blot analysis of phospho-SMAD3 (**d**) in HLF treated with increasing doses of CSF3 (50-200 ng/ml, 24 h). **e,** qRT-PCR of TGF-β1, IL-11, connective tissue growth factor (CTGF), and CSF3 expression in HLF treated with CSF3 (200 ng/ml, 24h). **f,** qRT-PCR analysis of TGF-β1 expression according to FB-101 treatment dose-dependent manner in IPDF. **g-i,** qRT-PCR analysis of TGF-β1 expression (**g**), Western blot analysis of phospho-SMAD3 expression (**h**), and ELISA quantification of TGF-β1 levels (**i**) in MLF and BLM-MLF treated with IgG or FB-101m. Statistical significance was determined using ANOVA with multiple comparison or t-test.

**
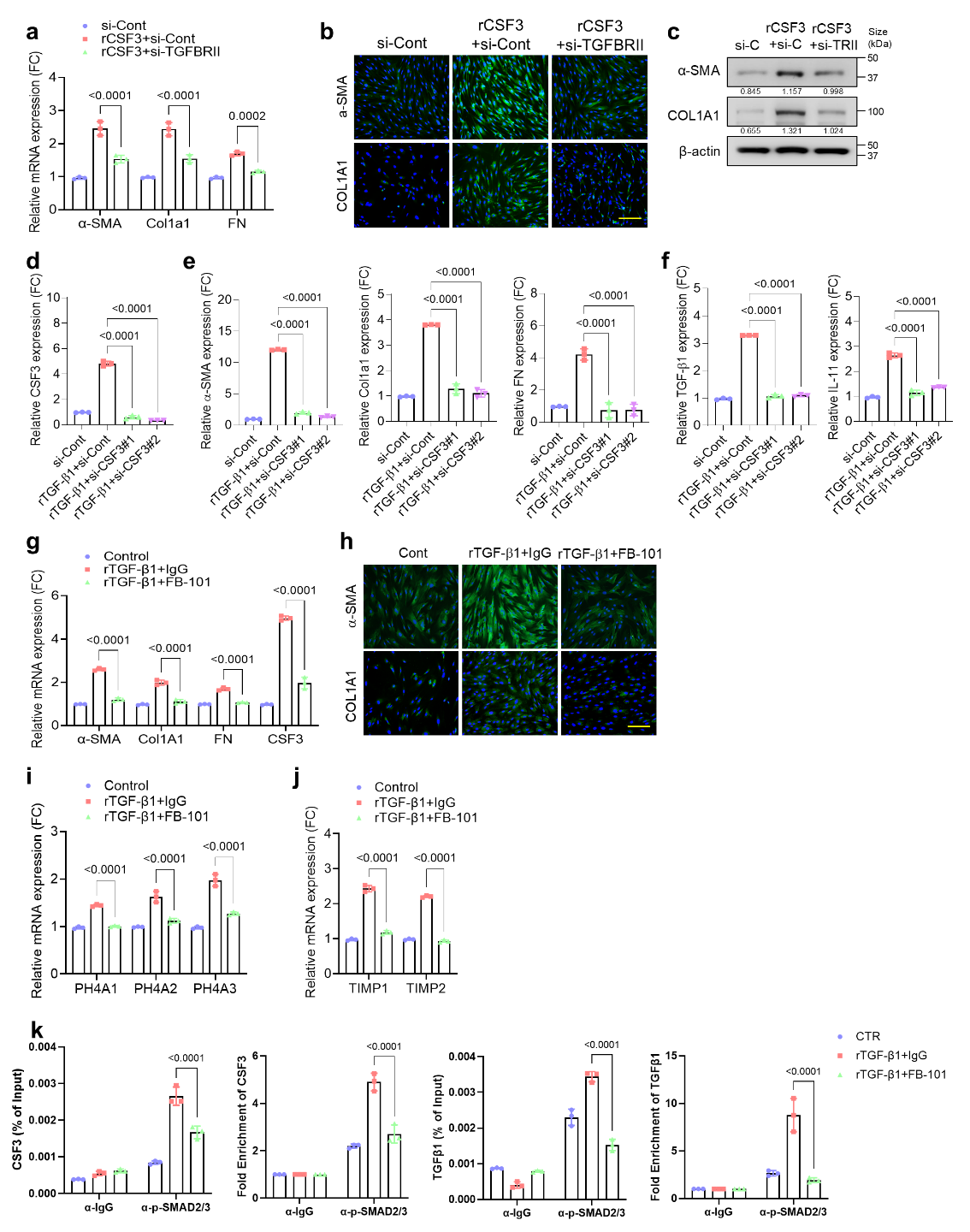
**

**Supplementary Figure. 11.**

**a-c,** qRT-PCR analysis of fibrosis markers (**a**), representative immunofluorescence images of α-SMA and COL1A1 expression (**b**), and Western blot analysis of α-SMA and COL1A1 expression (**c**) in HLF transfected with siRNA targeting TGFβRII and treated rCSF3 (200 ng/ml, 24 h). Scale bars: 200 μm. β-Actin was used as a loading control. **d-f,** qRT-PCR analysis of CSF3 (**d**), fibrosis markers (**e**), TGFβ1, and IL-11 (**f**) expression in HLF transfected with CSF3 siRNA and subsequently treated with recombinant TGF-β1 (rTGF-β1) (2 ng/ml, 24 h). **g-j,** qRT-PCR analysis of fibrosis markers and CSF3 expression (**g**), representative immunofluorescence images of α-SMA and COL1A1 immunostaining (**h**), qRT-PCR analysis of the prolyl hydroxylases (P4HA1, P4HA2, P4HA3) (**i**), and tissue inhibitors of metalloproteinases (TIMP1/2) (**j**) in HLF treated with rTGF-β1 and FB-101 as indicated. Scale bars: 200 μm. **k.** ChIP-qPCR analysis showed that phospho-SMAD2/3 can directly bind to the specific site (CGTCTAGACA) on the CSF3 and TGF-β1 promoters, which was confirmed to be regulated by FB-101. Statistical significance was determined using ANOVA with multiple comparison.

**
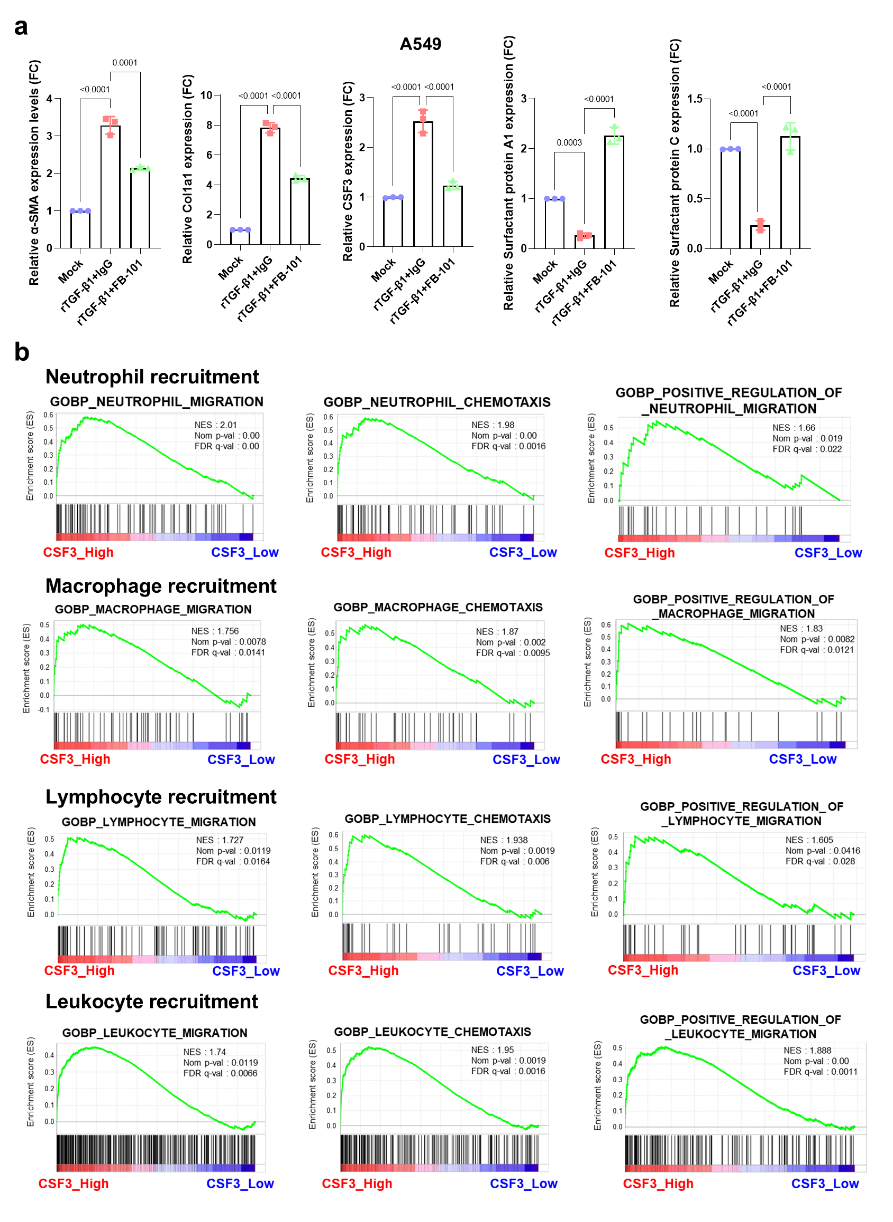
**

**Supplementary Figure. 12.**

**a,** qPCR analysis of A549 (AT2, alveolar cell type II phenotype lung cancer cell) treated with rTGF-β1 and FB-101 **b,** GSEA for neutrophils, macrophages, lymphocytes, and leukocytes recruitment-related signature in high/low CSF3 expression (GSE10667).

**Supplementary Table 1.** Primer list used in this study

| **Primer name (human)** |  | **5'- Oligo Seq -3'** |
| --- | --- | --- |
| 18S rRNA | F | ACC CGT TGA ACC CCA TTC GTG A |
|  | R | GCC TCA CTA AAC CAT CCA ATC GG |
| αSMA | F | CTA TGC CTC TGG ACG CAC AAC T |
|  | R | CAG ATC CAG ACG CAT GAT GGC A |
| Col1A1 | F | CAG ACT GGC AAC CTC AAG AA |
|  | R | CAG TGA CGC TGT AGG TGA AG |
| Col4A1 | F | CGG GCC CTA AAG GAG ATA AAG |
|  | R | GAA CCT GGA AAC CCA GGA AT |
| Fibronectin | F | GTG TAG CAC AAC TTC CAA TTA CGA A |
|  | R | GGA ATT TCC GCC TCG AGT CT |
| CSF3 | F | GCT GCT TGA GCC AAC TCC AT |
|  | R | CAT TCC CAG TTC TTC CAT CTG CT |
| TGF-β1 | F | CCC AGC ATC TGC AAA GCT C |
|  | R | GTC AAT GTA CAG CTG CCG CA |
| IL-11 | F | GGC TGC AGC TCC TGA TGT C |
|  | R | GAG TCT TCA GCA GCA GCA GT |
| TIMP1 | F | GGG ACA CCA GAA GTC AAC CA |
|  | R | GTT GTG GGA CCT GTG GAA GT |
| TIMP2 | F | TTG CAG GAG GAA TCG GTG AG |
|  | R | CAG GCA AGA AGC AAT GGC AA |
| MMP2 | F | TGC TGA AGG ACA CAC TAA AG |
|  | R | GTA GCC AAT GAT CCT GTA TGT |
| CTGF | F | ACC AAT GAC AAC GCC TCC T |
|  | R | TTG GAG ATT TTG GGA GTA CGG ATG |
| P4HA1 | F | CTG GAA AAT TGA CCA CAG CAC AG |
|  | R | CGT GCA AAG TCA AAA TGG GGT T |
| P4HA2 | F | CCG GGT TTC CAA AAG CTC CT |
|  | R | ACA CGA TTC CCC GTC CCT AA |
| P4HA3 | F | GTG GTG AAC TAT GGC ATC GGA |
|  | R | GTT CCA CCA AAA CAG TGC TGC |
| PLOD1 | F | GGG AGA GAC TTC ACC GTC CT |
|  | R | AGC GCC TGG ATC TTG TAG TT |
| PLOD2 | F | GAG AAG CCC TCG AGC ATC C |
|  | R | TTC TGG CCC CCT CCA ATA CT |
| PLOD3 | F | TCA ACT ACA CTG TGC GGA CC |
|  | R | CTG CCA CTC TGG ACG AAC TT |
| ChIP-p-STAT3-CSF3 | F | GCC CCC AAG CAC CAG AGA TG |
|  | R | GGC CTG AAC GTT TTC TAC AGG C |
| ChIP-p-STAT3- TGF-β1 | F | GCC TGG GGT CTC CAG TGA GTA |
|  | R | GGG CTA CTG GGC ACA TGG C |
| ChIP-p-SMAD2/3-CSF3 | F | TGG TCC GTG CCT GTG GT |
|  | R | CAT GTT GGC CAG GCT GGT |
| ChIP-p-SMAD2/3- TGF-β1 | F | CTT AAT GAA GCT TCC TTG ATC C |
|  | R | ATC CCT TCT CCT GTG CTA G |

| **Primer name (mouse)** |  | **5'- Oligo Seq -3'** |
| --- | --- | --- |
| αSMA | F | GTC CCA GAC ATC AGG GAG TAA |
|  | R | TCG GAT ACT TCA GCG TCA GGA |
| Col1A1 | F | CAC CCT CAA GAG CCT GAG TC |
|  | R | GTT CGG GCT GAT GTA CCA GT |
| Fibronectin | F | GTG TAG CAC AAC TTC CAA TTA CGA A |
|  | R | GGA ATT TCC GCC TCG AGT CT |
| CSF3 | F | AGC AGA CAC AGT GCC TAA GC |
|  | R | AGG TTT TCC ATC TGC TGC CAG A |
| TGF-β1 | F | TGA TAC GCC TGA GTG GCT GTC T |
|  | R | CAC AAG AGC AGT GAG CGC TGA A |
| TIMP1 | F | CAG TAA GGC CTG TAG CTG TGC |
|  | R | AGG TGG TCT CGT TGA TTT CTG |
| TIMP2 | F | GGA ATG ACA TCT ATG GCA ACC CCA |
|  | R | GGC CGT GTA GAT AAA CTC GAT GTC |
| MMP2 | F | ATG GAC CCC GGT TTC CCT AA |
|  | R | GGC TGC TTC ACA TCC TTC AC |
| CTGF | F | GAG TGT GCA CTG CCA AAG AT |
|  | R | GGC AAG TGC ATT GGT ATT TG |
| P4HA1 | F | CAT CCT GGC CCC AGC CAA GC |
|  | R | GGT GGC TCG CCT CAG CCT TG |
| P4HA2 | F | GTC CTG GTT CGG TGT CCT GAG C |
|  | R | CAG ATC GGT CAT GTG CCC AAT GG |
| P4HA3 | F | AGG CCC AAC GTA CCC CAC CT |
|  | R | GTG TGT TGG CTG GGA GCC CA |
| PLOD1 | F | ATG AGC AGC CTT CCT TGA TG |
|  | R | ATG AGC AGC CTT CCT TGA TG |
| PLOD2 | F | TGT TTA CCG AGT GTT TTG ATG T |
|  | R | ACG GGG CAT AGC CAA TAA AG |
| PLOD3 | F | AAT GCT GTC AAC CCA GAC AAA |
|  | R | AGT GTA GTT AAA GAA CTC CGC A |
