# Supplementary figures and images for: Colony-stimulating factor 3 as a key mediator in the progression of idiopathic pulmonary fibrosis: a novel therapeutic target
Source: Signal Transduct Target Ther. 2025 Oct 2;10:322. doi: 10.1038/s41392-025-02421-6 (PMC12489044; doi:10.1038/s41392-025-02421-6)

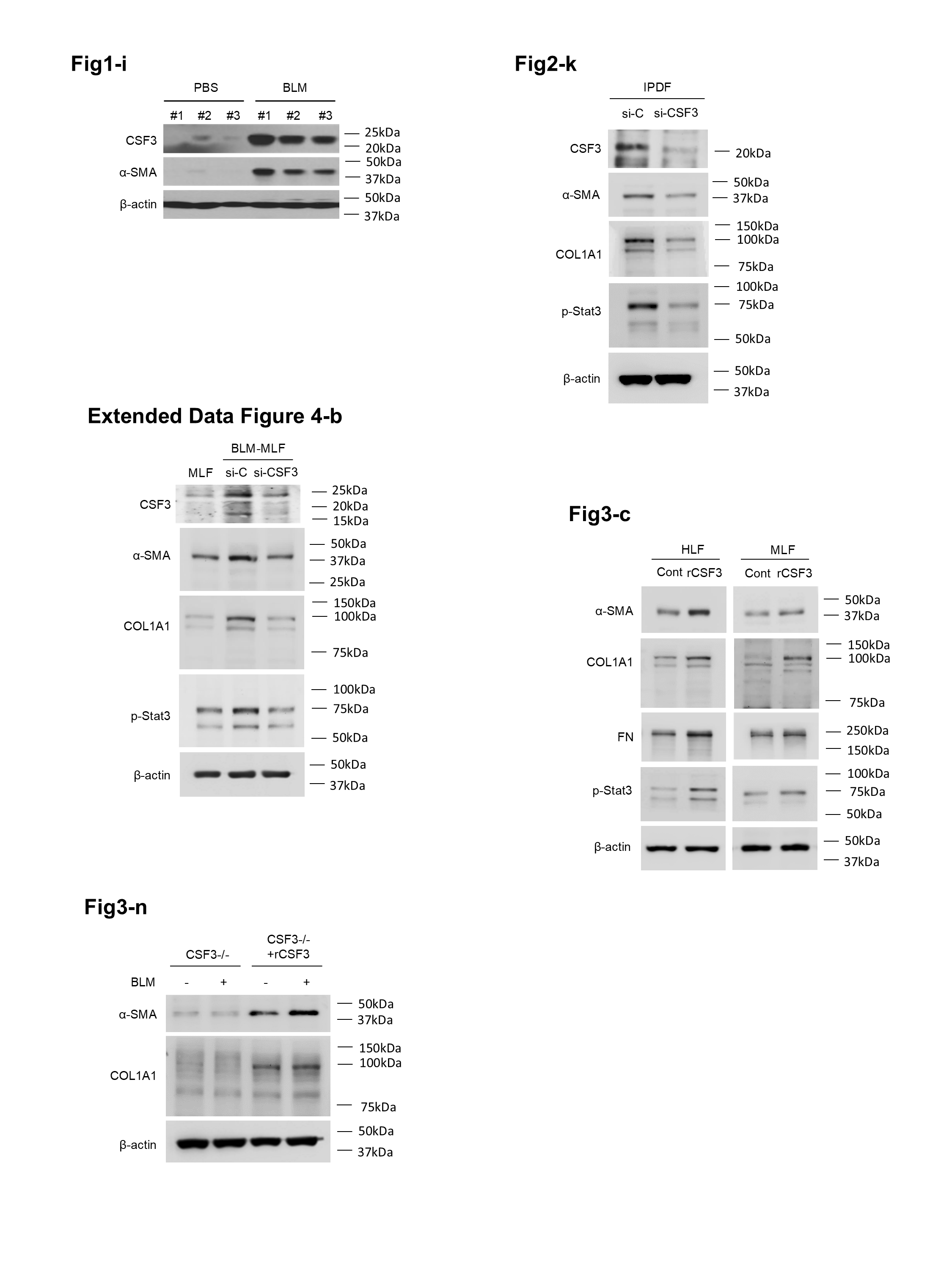

Supplement: Supplementary file 2 — Western blot Raw data - 1 [file 41392_2025_2421_MOESM2_ESM.tif]

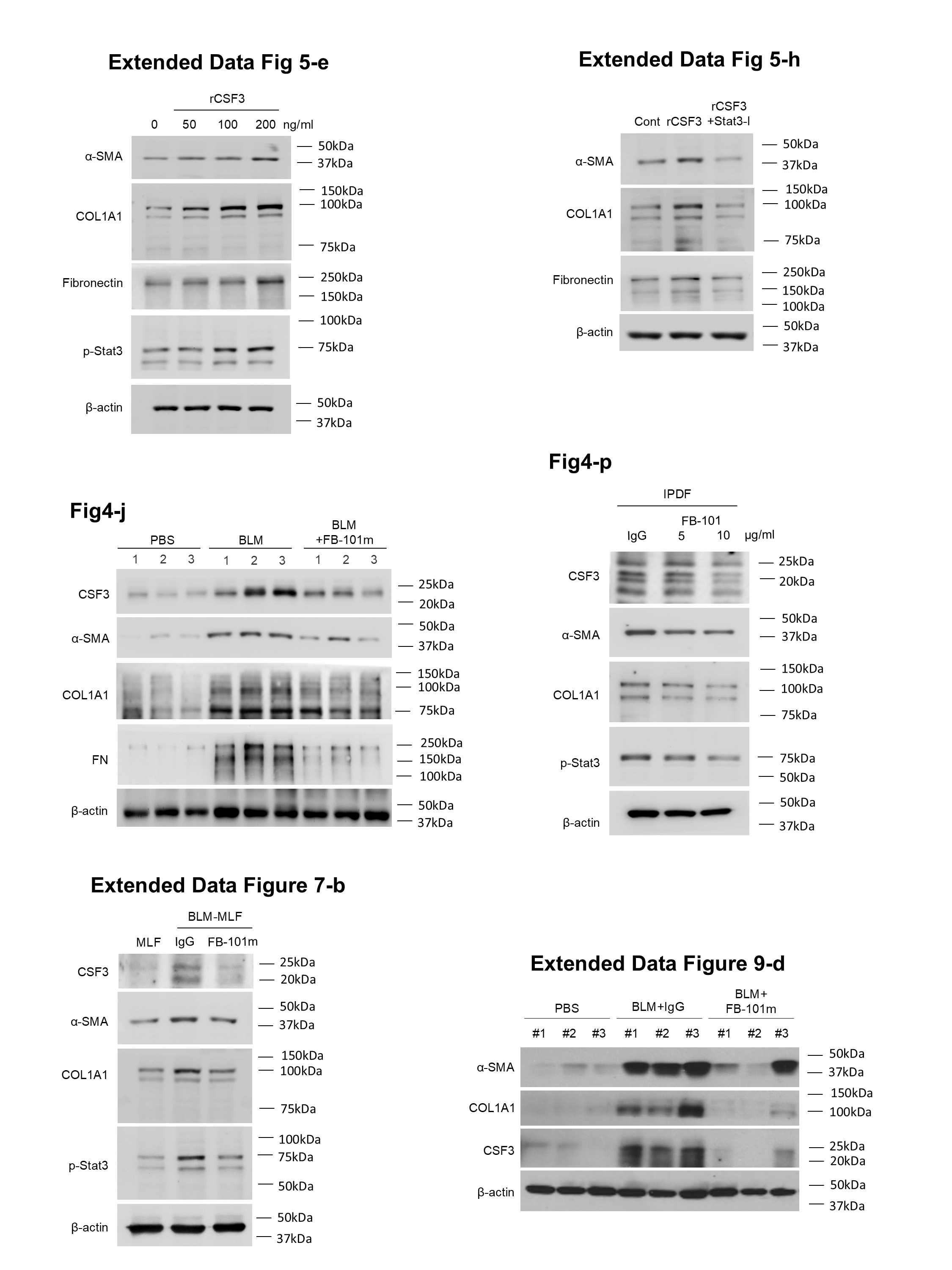

Supplement: Supplementary file 3 — Western blot Raw data - 2 [file 41392_2025_2421_MOESM3_ESM.tif]

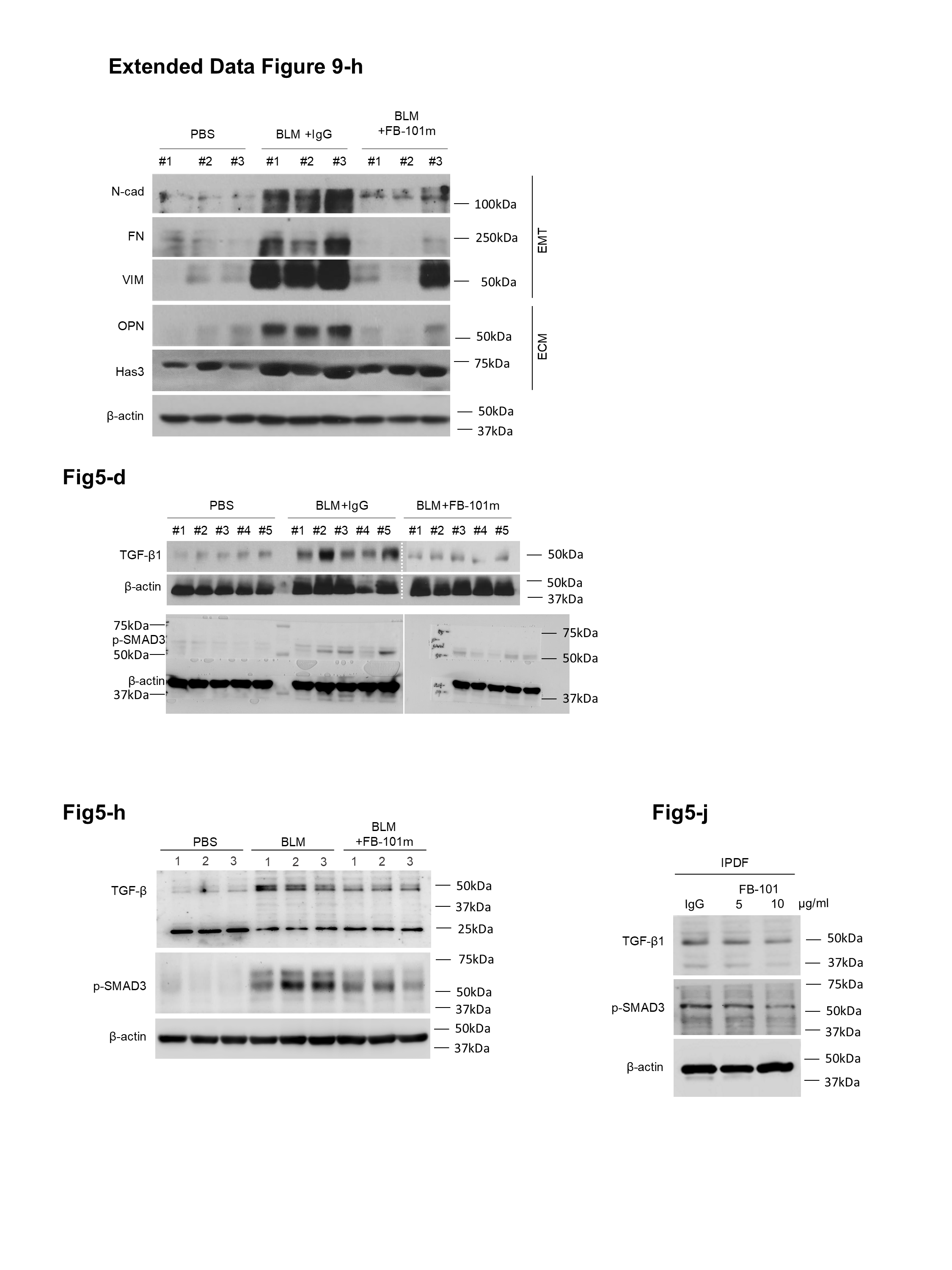

Supplement: Supplementary file 4 — Western blot Raw data - 3 [file 41392_2025_2421_MOESM4_ESM.tif]

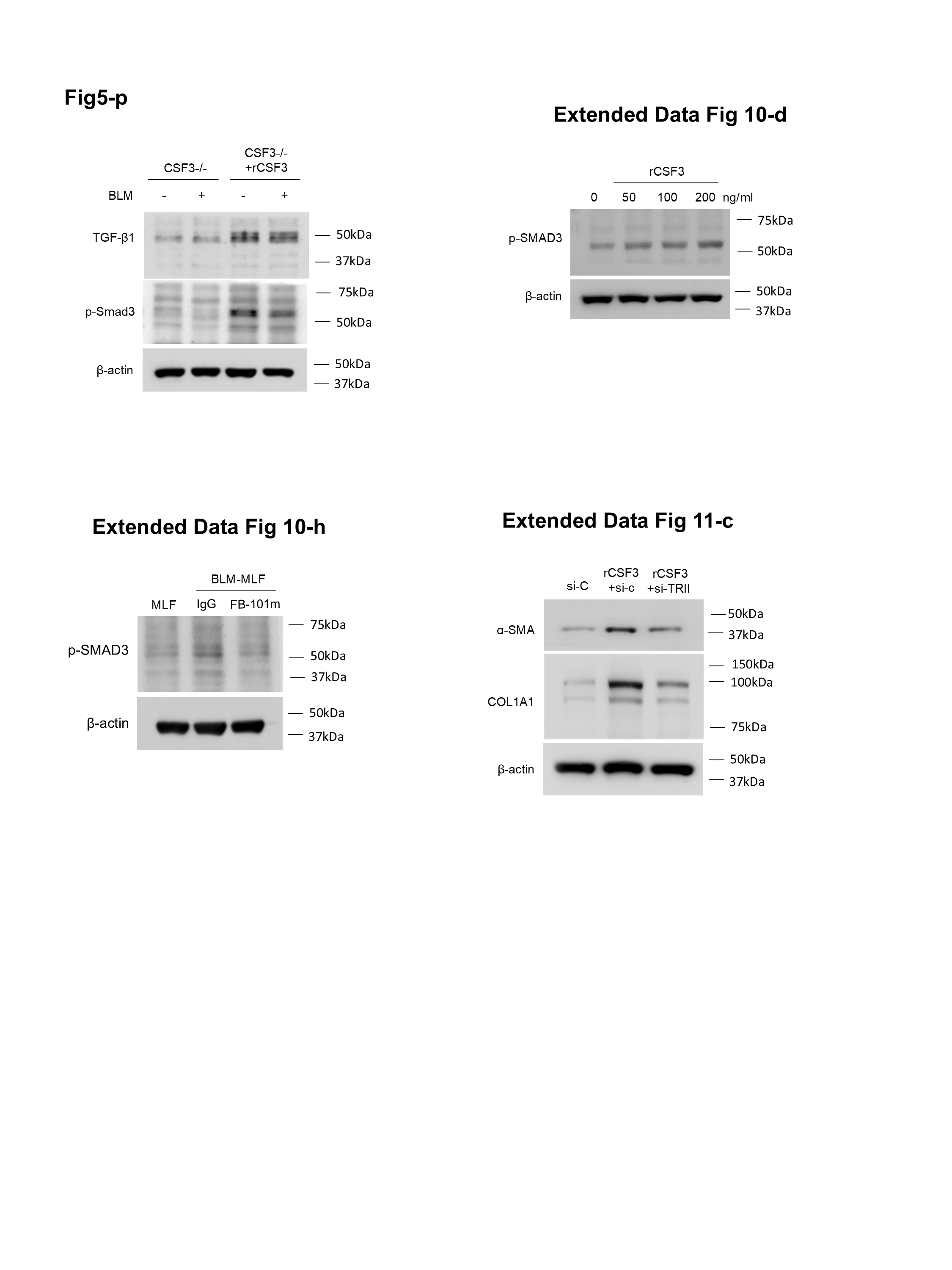

Supplement: Supplementary file 5 — Western blot Raw data - 4 [file 41392_2025_2421_MOESM5_ESM.tif]
